# When a Side Reaction Is a Benefit: A Catalyst-Free Route to Obtain High-Molecular Cobaltocenium-Functionalized Polysiloxanes by Hydroamination

**DOI:** 10.3390/polym16202887

**Published:** 2024-10-14

**Authors:** Anastasia N. Kocheva, Konstantin V. Deriabin, Igor Perevyazko, Nadezhda A. Bokach, Vadim P. Boyarskiy, Regina M. Islamova

**Affiliations:** 1 Institute of Chemistry, St. Petersburg State University, 7/9 Universitetskaya Nab., 199034 St. Petersburg, Russia; kocheva-nastya@mail.ru (A.N.K.);; 2 Department of Molecular Biophysics and Polymer Physics, St. Petersburg State University, 7/9 Universitetskaya Nab., 199034 St. Petersburg, Russia; igor.perevyazko@gmail.com

**Keywords:** polysiloxanes, cobaltocenium, hydroamination, molecular weight, redox properties

## Abstract

Cobaltocenium-containing (co)polysiloxanes (Cc-PDMSs) with terminal and side groups were synthesized by the reaction of catalyst-free hydroamination between ethynylcobaltocenium hexafluorophosphate and polysiloxanes comprising amino moieties as terminal and side groups. The conversion of NH_2_ groups in the polymers reaches 85%. The obtained (co)polysiloxanes “gelate” due to an increase in their molecular weight by approx. 30 times, when stored at room temperature over one week. “Gelated” Cc-PDMSs remain soluble in most polar solvents. The structure of Cc-PDMSs and the mechanism of “gelation” were established by ^1^H, ^13^C{^1^H}, ^29^Si{^1^H}, ^19^F{^1^H}, ^31^P{^1^H} nuclear magnetic resonance, infrared, ultraviolet–visible, and X-ray photoelectron spectroscopies. As determined by cyclic voltammetry, Cc-PDMSs possess redox properties (Co^II^/Co^III^ transitions at *E*_1/2_ = −1.8 and −1.3 V before and after “gelation”, respectively). This synthetic approach allows to increase the molecular weights of the synthesized polysiloxanes functionalized with cobaltocenium groups easily, leading to their higher film-forming ability, which is desirable for some electronic applications. Cc-PDMSs can be utilized as redox-active polymer films in modified electrodes, electrochromic devices, redox-active coatings, and components for batteries.

## 1. Introduction

The development of redox-active but highly flexible, soft, thermally stable, gas-permeable, and bioinert polymer materials with a low glass transition temperature is one of the major focus areas for flexible electronics and optoelectronics, soft robotics, stretchable electrochromic devices, etc. [1,2,3]. Functionalized (co)polysiloxanes fully meet all the above requirements in comparison with other prominent carbon-chain polymers [4,5].

The well-known ferrocenyl-containing (co)polysiloxanes and their composites with carbon nanotubes can be applied as flexible and stretchable modified electrodes in optoelectronics [3,6,7], neuronal implants in biomedicine [3], biosensors [8], electrochromic smart windows/mirrors [7], and antistatic coatings [6]. Polysiloxanes with ferrocenyl fragments in the main chain are synthesized preferably by ring-opening polymerization of ferrocenophanes or by polycondensation of difunctionalized ferrocene derivatives containing hydroxyl, carboxyl, or amino groups [3,9,10]. Copolysiloxanes with ferrocenyl pendant groups can be obtained by hydrosilylation, dehydrocoupling, azide–alkyne cycloaddition, hydrothiolation reactions, and anionic ring-opening polymerization of ferrocenylcyclosiloxanes [3,6,7,11,12,13]. However, these reactions predominantly proceed in the presence of either initiators or catalysts, such as azobisisobutyronitrile, silanoates, or metal complexes, so it is necessary to purify the resulting products from foreign impurities.

For the present, there are still no suitable alternatives to these redox-active polysiloxanes. In this regard, some of the most promising redox-active silicones are cobaltocenium-containing polysiloxanes [14,15]. Cobaltocenium is a chemically stable compound that has two redox transitions in a negative region at −1.3 and −2.2 V as distinguished from the ferrocene with Fc/Fc^+^ redox couple at approx. +0.4 V [7,16]. Due to the redox activity and positive charge of cobaltocenium, cobaltocenium-containing polymers are used as anion-exchange membranes [14], electrosorbents [17], sensors [1,14], electrocatalysts [14,18], antimicrobial hydrogels [19], and antibiotic bioconjugates [20]. Furthermore, cobaltocenium-containing polysiloxanes would have many promising applications as modified electrodes [1,12,21], multicolor electrochromic devices [1,7,22], redox-active coatings [6,15,23,24], corrosion inhibitors [25,26], stimuli-responsive materials and actuators [1,27], and as components for batteries containing redox-active polymer films [1,28].

Currently, only a few cobaltocenium-containing polysiloxanes and one disiloxane have been obtained by the acylation of amino groups [14,21]. The amide-linked cobaltocenium groups were incorporated into the polymer backbone either as a part of the main polymer chain or as pendant side groups. The obtained products were characterized by ^1^H, ^13^C, ^29^Si nuclear magnetic resonance (NMR) spectroscopy, Fourier-transform infrared (FTIR) spectroscopy, elemental analysis, and cyclic voltammetry (CV). The polymer with main chain cobaltocenium moieties was essentially insoluble in most common organic solvents except DMSO, so its molecular weight was unknown. The polysiloxane with pendant cobaltocenium groups had the number-average molecular weight of 4900, determined by vapor-phase osmometry.

In general, the known cobaltocenium-containing polysiloxanes have poor solubility in most common solvents and relatively low molecular weights. However, the high molecular weight of a polymer generally provides its good film-forming ability, which is desirable for practical applications as components of batteries and other electronic and optoelectronic devices [21,27,28]. It should be noted that high molecular weights of functionalized polysiloxanes can be achieved in most cases by the labor-intensive multistage synthetic routes (for example, catalytic modification of polymer chains or polycondensation) [1,14,15,21]. In this regard, we hypothesized that the development of a catalyst-free hydroamination reaction can result in high-molecular-weight cobaltocenium-containing (co)polysiloxanes [29,30,31]. Noteworthily, many hydroamination reactions can tolerate various functional groups and generally involve fewer by-products than traditional methods, allowing the synthesis of a wide range of compounds, which is important for functionalized polymer synthesis. Therefore, we used a polysiloxane containing amino groups (either in the side chain or terminal) as the amino component in this reaction. We believe that the simplest “gelation” after the hydroamination can act as a benefit of the process since it leads to an increase in the molecular weight of cobaltocenium-containing polysiloxanes. Here, we present the results of this study.

## 2. Materials and Methods

### 2.1. Materials

*α*,*ω*-Di((3-aminopropyl)dimethylsiloxy)polydimethylsiloxanes (APDMSs) with number-average molecular weights of *M_n_* = 850–900 (APDMS850, viscosity 10–15 cSt), *M_n_* = 5000 (APDMS5000, viscosity 100–120 cSt), and *M_n_* = 25,000 (APDMS25000, viscosity 900–1100 cSt) were purchased from Abcr GmbH (Karlsruhe, Germany) and used as received. 1,3-Bis(3-aminopropyl)tetramethyldisiloxane (APTMDS, 97%) and poly((3-aminopropyl)methylsiloxane-*co*-dimethylsiloxane) with 2–3 mol.% of (3-aminopropyl)-containing siloxane units (P(AMS-*co*-DMS), viscosity 80–200 cSt) were also obtained from Abcr GmbH (Karlsruhe, Germany) and used without additional purification. Et_4_NBF_4_ (≥99%, Merck, St. Louis, MO, USA) was recrystallized from an azeotropic *i*-PrOH/H_2_O mixture (87.5 wt.%, NevaReaktiv, St. Petersburg, Russia) and then from CH_3_CN for the CV tests. CH_2_Cl_2_ (99%) and CH_3_CN (99%) were purchased from Vekton (St. Petersburg, Russia) and distilled over P_4_O_10_ (98%, Component-Reaktiv, Moscow, Russia) under argon prior to use. Toluene (99%) and acetone (99%) were bought from NevaReactiv (St. Petersburg, Russia) and used as received. Ethynylcobaltocenium hexafluorophosphate was synthesized from cobaltocene according to the well-known four-step procedure [32] and fully characterized by NMR spectroscopy in acetone-*d*_6_ before usage (Figure S1, Supplementary Material).

### 2.2. Methods

#### 2.2.1. Spectroscopy Equipment and Studies

The NMR spectra were recorded on a Bruker Avance III 400 spectrometer (Bruker, Billerica, MA, USA) in CDCl_3_ and acetone-*d*_6_ at room temperature (r.t., 23 °C) operating at 400, 101, 80, 376, and 162 MHz for ^1^H, ^13^C, ^29^Si, ^19^F, and ^31^P nuclei, respectively. The chemical shifts of the signals are shown in *δ*-values [ppm] referenced to the residual signals of non-deuterated solvents: CHCl_3_ (*δ* = 7.26) and acetone (*δ* = 2.05) in the case of ^1^H, to the signals of deuterated solvents: CDCl_3_ (*δ* = 77.2) and acetone-*d*_6_ (*δ* = 29.8) in the case of ^13^C{^1^H}, as well as signals of external standards (CH_3_)_4_Si, CFCl_3_, and H_3_PO_4_ in the case of ^29^Si{^1^H}, ^19^F{^1^H}, and ^31^P{^1^H} NMR spectroscopy, respectively. The following abbreviations were used to designate multiplicities: s = singlet, d = doublet, t = triplet, q = quartet, p = pentet, sxt = sextet, h = heptet, m = multiplet, br. = broad, dd = doublet of doublets, dt = doublet of triplets, td = triplet of doublets, qd = quartet of doublets.

The FTIR spectra were obtained using the attenuated total reflection technique (FTIR-ATR) on a Thermo Scientific Nicolet 8700 FTIR spectrophotometer (Thermo Fisher Scientific, Waltham, MA, USA) with a DTGS detector in the range of 400–4000 cm^−1^ at r.t. Apodization and phase correction were performed for the FTIR-ATR spectra using the Blackman–Harris function and the Mertz method, respectively. The following abbreviations of the absorption bands are utilized to designate intensity: st—strong, md—medium, wk—weak.

The ultraviolet–visible (UV–vis) spectra of polymers were recorded by using their solutions in distilled CH_3_CN (concentration 16 μm·mL^−1^) on a Shimadzu UV-1800 spectrophotometer (Shimadzu, Kyoto, Japan). Measurements were carried out at r.t. using a quartz cuvette 1 cm wide in the wavelength range of 200–800 nm.

High-resolution mass spectra (HRMS) were recorded in a positive-ion mode (*m/z* = 100–1000) on a Bruker Maxis HRMS-ESI-qTOF spectrometer (Bruker, Billerica, MA, USA) that was equipped with an electrospray ionization source. The analyzed compounds were dissolved in pure CH_3_CN prior to the measurements. A molecular ion peak in the isotopic pattern was noted.

The cobalt content in polymer samples was determined by X-ray photoelectron spectroscopic (XPS) analysis of their surface. The XPS spectra were acquired using an Escalab 250Xi photoelectron spectrometer (Waltham, MA, USA) with AlKα radiation (photon energy 1486.6 eV). The spectra were recorded in the constant pass energy mode at 100 eV for the survey spectrum and 50 eV for the element core level spectrum, using an XPS spot size of 650 μm.

#### 2.2.2. Sedimentation Velocity Experiments

Sedimentation velocity experiments were performed with a ProteomeLab XLI Protein Characterization System analytical ultracentrifuge (Beckman Coulter, Brea, CA, USA) using double-sector cells with aluminum centerpieces with an optical path length of 12 mm, and a four-hole analytical rotor (An-60Ti) was used. The rotor speed was 55,000 rpm. A polymer sample and reference sectors were loaded using 0.43 mL of the studied solution and 0.45 mL of a solvent (acetone in the case of Cc-APDMS850, Cc-APDMS5000, and P(Cc-AMS-*co*-DMS); toluene in the case of Cc-APDMS25000), respectively. Sedimentation profiles were obtained using the interference optical system equipped with a red laser (*λ* = 655 nm) as a light source. The centrifuge chamber with a loaded rotor and interferometer was vacuumed and thermostatted at 25 °C for 1 h before the run. Prior to the sedimentation velocity experiments, a solvent was evaporated from the analyzed (co)polymers; the (co)polymers were dried and then stored in air for up to 3 months. The velocity sedimentation data analysis was processed using the Sedfit software (version 16.50, May 2023) [33].

#### 2.2.3. Cyclic Voltammetry

CV of the polymer solutions in dry CH_3_CN was conducted with different scan rates using a Biologic VMP3 potentiostat (BioLogic Science Instruments, Seyssinet-Pariset, France), in a classic three-electrode electrochemical cell with a septum and a constant gentle stream of dry argon. A Pt microelectrode (0.07 mm^2^), Pt metal band (15.0 cm^2^), and non-aqueous Ag|AgNO_3_ (MF-2062, Bioanalytical systems, West Lafayette, IN, USA, 100 mM LiClO_4_ and 5 mM AgNO_3_ in CH_3_CN internal solution; −0.125 V vs. Fc/Fc^+^) were used as the working, counter, and reference electrodes, respectively. A 0.1 M solution of Et_4_NBF_4_ in dry CH_3_CN was utilized as an electrolyte. Before the CV measurements, a gentle stream of argon was purged through the analyzed solution for 10 min. The CV tests were carried out with *iR* compensation (approx. 95%) at scan rates of 0.05–50 V·s^−1^ within the potential range from −1.8 to 0.0 V vs. Ag|AgNO_3_. After the CV measurements, ferrocene was added to the electrochemical cell as an internal standard. The potential values are given relative to the Fc/Fc^+^ potential.

### 2.3. Synthesis of Cobaltocenium-Containing Polysiloxanes

The cobaltocenium-containing polysiloxanes (Cc-PDMSs) were synthesized from the amino-containing polysiloxanes (APTMDS, APDMS850, APDMS5000, APDMS25000, P(AMS-*co*-DMS)) similar to the previously reported method [31] but with some modifications. Prior to synthesis, the method had been optimized by choosing temperatures, reaction times, and suitable CH_2_Cl_2_/CH_3_CN mixtures to dissolve all the reagents. Noteworthily, both shorter reaction times (than 48 h) and lower temperatures (than reflux at 40 °C) led to reduced conversions of the NH_2_ groups. Attempts to carry out hydroamination longer than 48 h (e.g., 72 h) led to the conversion of NH_2_ groups being close to the reaction for 48 h.

Thus, a calculated amount of an amino-containing polysiloxane (0.28 mmol of amino groups) was dissolved in a mixture of dry CH_3_CN and anhydrous CH_2_Cl_2_ in a round-bottom flask under an argon atmosphere (amounts of polymers, CH_3_CN, and CH_2_Cl_2_ are given in Table 1). Afterward, a solution of ethynylcobaltocenium hexafluorophosphate (100 mg, 0.28 mmol) in 1.5 mL of the CH_3_CN/CH_2_Cl_2_ mixture (in solvent ratios given in Table 1) was added to the solution of the amino-containing polysiloxane. The reaction mixture was then stirred at 40 °C under argon for 48 h. The solvent was removed under reduced pressure (200–600 mbar, 45 °C), and the target polymer product (Cc-APTMDS, Cc-APDMS850, Cc-APDMS5000, Cc-APDMS25000, or P(Cc-AMS-*co*-DMS)) was dried under reduced pressure (down to 5 mbar, 50 °C) for 1 h to remove traces of solvents. The number-average molecular weights (*M_n_*) of Cc-PDMSs were determined via ^1^H NMR end-group analysis by comparing the integrals of the signals corresponding to intrachain dimethylsiloxane units at 0.09–0.14 ppm and terminal groups. In the case of Cc-APDMSs, calculations were based on the signals at 3.05–3.14 ppm related to CH_2_CH_2_C*H*_2_NH terminal groups. For the copolysiloxane with pendant cobaltocenium moieties (P(Cc-AMS-*co*-DMS)), the determination of *M_n_* was based on the integral of the signal corresponding to the (C*H*_3_)_3_Si terminal groups at 0.08 ppm.

Cc-APTMDS (freshly synthesized). Yield: 125 mg (96%); conversion of NH_2_ groups is 100%; dark red oil. ^1^H NMR (acetone-*d*_6_, *δ*, ppm): 0.10 (s, C*H*_3_Si), 0.63 (m, 4H, SiC*H*_2_CH_2_CH_2_NH), 1.66 (p, *J* = 7.3 Hz, 4H, SiCH_2_C*H*_2_CH_2_NH), 3.11 (q, *J* = 6.0 Hz, 4H, SiCH_2_CH_2_C*H*_2_NH), 4.99 (d, *J* = 13.5 Hz, 2H, *trans*-CH_2_NHCH=C*H*Cc), 5.47 (s, 10H, Cp), 5.66 (m, 4H, 3,4-C_5_*H*_4_), 5.76 (m, 4H, 2,5-C_5_*H*_4_), 6.37 (br. s, 2H, CH_2_N*H*CH=CHCc), 7.47 (m, 2H, *trans*-CH_2_NHC*H*=CHCc). ^13^C{^1^H} NMR (acetone-*d*_6_, *δ*, ppm): 0.5 (*C*H_3_Si), 16.1 (Si*C*H_2_CH_2_CH_2_NH), 23.7 (SiCH_2_*C*H_2_CH_2_NH), 47.8 (SiCH_2_CH_2_*C*H_2_NH), 74.5 (2,5-*C_5_*H_4_), 82.3 (3,4-*C*_5_H_4_), 83.9 (CH_2_NHCH=*C*HCc), 85.3 (Cp), 117.9 (*ipso*-*C*_5_H_4_), 146.4 (CH_2_NH*C*H=CHCc). ^29^Si{^1^H} NMR (acetone-*d*_6_, *δ*, ppm): 7.8 ((CH_3_)_2_(CcCH=CHNHCH_2_CH_2_CH_2_)*Si*O–; M^Cc^). ^19^F{^1^H} NMR (acetone-*d*_6_, *δ*, ppm): −72.3 (d, *J* = 707.9 Hz, P*F*_6_^−^). ^31^P{^1^H} NMR (acetone-*d*_6_, *δ*, ppm): −144.2 (h, *J* = 708.1 Hz, *P*F_6_^−^). *M* = 965 (based on ^1^H NMR). HRMS^+^: calculated for [C_34_H_48_Co_2_N_2_OSi_2_]^2+^ 337.0984, found *m*/*z* 337.0977 [M]^2+^.

Cc-APDMS850 (freshly synthesized). Yield: conversion of NH_2_ groups is 85%; viscous dark red oil, which solidifies when stored at r.t. ^1^H NMR (acetone-*d*_6_, *δ*, ppm): 0.09 (br. s, terminal C*H*_3_Si), 0.09 and 0.12 (br. s, C*H*_3_Si), 0.65 (m, 4H, SiC*H*_2_CH_2_CH_2_NH), 1.67 (m, 4H, SiCH_2_C*H*_2_CH_2_NH), 3.13 (qd, *J*_1_ = 6.5 Hz, *J*_2_ = 4.0 Hz, 4H, SiCH_2_CH_2_C*H*_2_NH), 4.99 (d, *J* = 13.6 Hz, 2H, *trans*-CH_2_NHCH=C*H*Cc), 5.47 (s, 10H, Cp), 5.67 (m, 4H, 3,4-C_5_*H*_4_), 5.77 (m, 4H, 2,5-C_5_*H*_4_), 6.43 (br. s, 2H, CH_2_N*H*CH=CHCc), 7.49 (dt, *J*_1_ = 13.6 Hz, *J*_2_ = 4.0 Hz, 2H, *trans*-CH_2_NHC*H*=CHCc). ^13^C{^1^H} NMR (acetone-*d*_6_, *δ*, ppm): 0.4 (terminal *C*H_3_Si), 1.4 (*C*H_3_Si), 16.0 (Si*C*H_2_CH_2_CH_2_NH), 23.7 (SiCH_2_*C*H_2_CH_2_NH), 47.7 (SiCH_2_CH_2_*C*H_2_NH), 74.5 (2,5-*C*_5_H_4_), 82.4 (3,4-*C*_5_H_4_), 83.8 (CH_2_NHCH=*C*HCc), 85.3 (Cp), 117.9 (*ipso*-*C*_5_H_4_), 146.3 (CH_2_NH*C*H=CHCc). ^29^Si{^1^H} NMR (acetone-*d*_6_, *δ*, ppm): −21.9 (– [(CH_3_)_2_*Si*O]– units; DDD), −21.3 (–[(CH_3_)_2_*Si*O]– pre-terminal units; M^Cc^DD), 7.6 (terminal (CH_3_)_2_(CcCH=CHNHCH_2_CH_2_CH_2_)*Si*O–; M^Cc^D). ^19^F{^1^H} NMR (acetone-*d*_6_, *δ*, ppm): −72.4 (d, *J* = 707.7 Hz, P*F*_6_^−^). ^31^P{^1^H} NMR (acetone-*d*_6_, *δ*, ppm): −144.2 (h, *J* = 707.7 Hz, *P*F_6_^−^). *M_n_* = 1630 (based on ^1^H NMR). FTIR-ATR (selected bands, *ν*, cm^−1^): 3437 (wk; *ν*(N– H)), 3125 (wk, *ν*(C_Cp_– H)); 2963 (st; *ν*(C_CH3_– H) + *ν_as_*(C_CH2_– H)), 2904 (md; *ν_s_*(C_CH2_– H)), 1623 (st; *ν*(C=C)), 1258 (st; *ν*(Si– C)), 1013 (st; *ν*(Si– O)), 787 (st; *δ*(C– H)). UV–vis (CH_3_CN, *λ_max_*, nm): 415 (*d–d*), 481 (ligand-to-metal charge transfer (LMCT)).

Cc-APDMS5000 (freshly synthesized). Yield: conversion of NH_2_ groups is 77%; viscous dark red oil, which solidifies when stored at r.t. ^1^H NMR (acetone-*d*_6_, *δ*, ppm): 0.10 (br. s, terminal C*H*_3_Si), 0.14 (br. s, C*H*_3_Si), 0.67 (m, 5H, SiC*H*_2_CH_2_CH_2_NH), 1.69 (m, 5H, SiCH_2_C*H*_2_CH_2_NH), 3.14 (t, *J* = 7.1 Hz, 4H, SiCH_2_CH_2_C*H*_2_NH), 5.00 (d, *J* = 13.5 Hz, 1H, *trans*-CH_2_NHCH=C*H*Cc), 5.49 (s, 10H, *Cp*), 5.68 (br. td, *J* = 1.9 Hz, 4H, 3,4-C_5_*H*_4_), 5.78 (pseudo t, 4H, 2,5-C_5_*H*_4_), 6.48 (br. s, CH_2_N*H*CH=CHCc), 7.50 (d, *J* = 13.5 Hz, 2H, *trans*-CH_2_NHC*H*=CHCc). ^13^C{^1^H} NMR (acetone-*d*_6_, *δ*, ppm): 0.4 (terminal *C*H_3_Si), 1.4 (*C*H_3_Si), 16.1 (Si*C*H_2_CH_2_CH_2_NH), 23.9 (SiCH_2_*C*H_2_CH_2_NH), 47.7 (SiCH_2_CH_2_*C*H_2_NH), 74.4 (2,5-*C*_5_H_4_), 82.3 (3,4-*C*_5_H_4_), 83.8 (CH_2_NHCH=*C*HCc), 85.3 (Cp), 117.9 (*ipso*-*C*_5_H_4_), 144.7 (CH_2_NH*C*H=CHCc). ^29^Si{^1^H} NMR (acetone-*d*_6_, *δ*, ppm): −22.0 (– [(CH_3_)_2_*Si*O]– units; DDD), −21.3 (–[(CH_3_)_2_*Si*O]– pre-terminal units; M^Cc^DD), 7.6 (terminal (CH_3_)_2_(CcCH=CHNHCH_2_CH_2_CH_2_)*Si*O–; M^Cc^D). ^19^F{^1^H} NMR (acetone-*d*_6_, *δ*, ppm): −72.5 (d, *J* = 707.7 Hz, P*F*_6_^−^). ^31^P{^1^H} NMR (acetone-*d*_6_, *δ*, ppm): −144.2 (h, *J* = 707.7 Hz, *P*F_6_^−^). *M_n_* = 5860 (based on ^1^H NMR).

Cc-APDMS25000 (freshly synthesized). Yield: conversion of NH_2_ groups is 45%; viscous red oil, which solidifies when stored at r.t. ^1^H NMR (CDCl_3_, *δ*, ppm): 0.07 (br. s, C*H*_3_Si), 0.14 (br. s, terminal C*H*_3_Si), 0.56 (m, 4.8H, SiC*H*_2_CH_2_CH_2_NH), 1.26 (br. s, unreacted NH_2_), 1.48 (p, *J* = 7.3 Hz, 2.1H, unreacted SiCH_2_C*H*_2_CH_2_NH_2_), 1.62 (m, 2.2H, SiCH_2_C*H*_2_CH_2_NHCH=CHCc), 2.69 (t, *J* = 7.3 Hz, 2.2H, unreacted SiCH_2_CH_2_C*H*_2_NH_2_), 3.05 (q, *J* = 7.0 Hz, 1.8H, SiCH_2_CH_2_C*H*_2_NHCH=CHCc), 3.32 (s, unreacted CcC≡C*H*), 4.75 (d, *J* = 13.6 Hz, 0.8H, *trans*-CH_2_NHCH=C*H*Cc), 5.00 (br. s, 0.9H, CH_2_N*H*CH=CHCc), 5.34 (s, 4.7H, Cp), 5.46 (br. m, 1.8H, 3,4-C_5_*H*_4_), 5.56 (br. pseudo t, 1.8H, 2,5-C_5_*H*_4_), 5.79 (m, unreacted 3,4-C_5_*H*_4_CpCoC≡CH), 5.80 (s, unreacted *Cp*CoC_5_H_4_C≡CH), 5.91 (m, unreacted 2,5-C_5_*H*_4_CpCoC≡CH), 7.43 (dd, *J*_1_ = 13.6 Hz, *J*_2_ = 7.0 Hz, 0.9H, *trans*-CH_2_NHC*H*=CHCc). ^13^C{^1^H} NMR (acetone-*d*_6_, *δ*, ppm): 0.3 (terminal *C*H_3_Si), 1.2 (*C*H_3_Si), 73.4 (2,5-*C*_5_H_4_), 81.0 (3,4-*C*_5_H_4_), 84.5 (Cp), 85.9 (unreacted 2,5-*C*_5_H_4_CpCoC≡CH), 86.0 (unreacted 3,4-*C*_5_H_4_CpCoC≡CH), 86.7 (unreacted *Cp*CoC_5_H_4_C≡CH). ^29^Si{^1^H} NMR (CDCl_3_, *δ,* ppm): −22.0 (– [(CH_3_)_2_*Si*O]– units; DDD), 7.6 (terminal (CH_3_)_2_(CcCH=CHNHCH_2_CH_2_CH_2_)*Si*O–; M^Cc^D). *M_n_* = 23,150 (based on ^1^H NMR).

P(Cc-AMS-*co*-DMS) (freshly synthesized). Yield: conversion of NH_2_ groups is 67%; viscous dark red oil, which solidifies when stored at r.t. ^1^H NMR (acetone-*d*_6_, *δ*, ppm): 0.08 (br. s, terminal C*H*_3_Si), 0.09 and 0.13 (br. s, C*H*_3_Si), 0.65 (m, 3.4H, SiC*H*_2_CH_2_CH_2_NH), 1.72 (m, 3.0H, SiCH_2_C*H*_2_CH_2_NH), 3.14 (t, *J* = 5.6 Hz, 2.0H, SiCH_2_CH_2_C*H*_2_NHCH=CHCc), 3.28 (br. m, 1.0H, unreacted SiCH_2_CH_2_C*H*_2_NH_2_), 4.16 (s, unreacted CcC≡C*H*), 5.00 (d, *J* = 13.5 Hz, 1.0H, *trans*-CH_2_NHCH=C*H*Cc), 5.49 (s, 5H, Cp), 5.69 (m, 2H, 3,4-C_5_*H*_4_), 5.78 (m, 2H, 2,5-C_5_*H*_4_), 5.99 (s, unreacted *Cp*CoC_5_H_4_C≡CH), 6.00 (m, unreacted 3,4-C_5_*H*_4_CpCoC≡CH), 6.20 (pseudo t, 2,5-C_5_*H*_4_CpCoC≡CH), 6.49 (br. s, CH_2_N*H*CH=CHCc), 7.51 (d, *J* = 13.5 Hz, 1.0H, *trans*-CH_2_NHC*H*=CHCc). ^13^C{^1^H} NMR (acetone-*d*_6_, *δ*, ppm): 0.9, 1.0, 1.2, 1.4, 1.8, and 2.0 (*C*H_3_Si), 15.5 (Si*C*H_2_CH_2_CH_2_NH), 23.3 (SiCH_2_*C*H_2_CH_2_NH), 47.5 (SiCH_2_CH_2_*C*H_2_NHCH=CHCc), 54.1 (SiCH_2_CH_2_*C*H_2_NH_2_), 74.5 (2,5-*C*_5_H_4_), 82.4 (3,4-*C*_5_H_4_), 85.3 (Cp), 86.0 (CH_2_NHCH=*C*HCc), 86.1 (unreacted 2,5-*C*_5_H_4_CpCoC≡CH), 87.6 (unreacted *Cp*CoC_5_H_4_C≡CH), 87.7 (unreacted 3,4-*C*_5_H_4_CpCoC≡CH). ^29^Si{^1^H} NMR (acetone-*d*_6_, *δ*, ppm): −22.1 (– [(CH_3_)(RNHCH_2_CH_2_CH_2_)*Si*O]– units, R = H, CcCH=CH; DD^X^D, X = Cc, NH_2_)), −22.0 (– [(CH_3_)_2_*Si*O]– units; DDD), −21.6 (– [(CH_3_)_2_*Si*O]– pre-terminal units; MDD), 7.2 (terminal (CH_3_)_3_*Si*O–; MD). ^19^F{^1^H} NMR (acetone-*d*_6_, *δ*, ppm): −72.5 (d, *J* = 707.6 Hz, P*F*_6_^−^). ^31^P{^1^H} NMR (acetone-*d*_6_, *δ*, ppm): −144.3 (h, *J* = 707.6 Hz, *P*F_6_^−^). *M_n_* = 7930 (based on ^1^H NMR).

## 3. Results and Discussion

### 3.1. Synthesis and Characterization of Cobaltocenium-Containing Polysiloxanes

**Figure 1:**
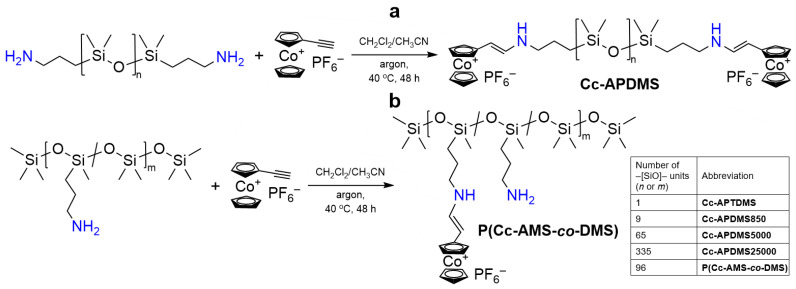
Synthetic schemes for Cc-APDMSs (**a**) and P(Cc-AMS-*co*-DMS) (**b**).

Cobaltocenium-terminated polydimethylsiloxanes (Cc-APDMSs) were synthesized by the reaction between pre-prepared ethynylcobaltocenium hexafluorophosphate and APDMSs with different number-average molecular weights of *M_n_* = 248, 850, 5000, and 25,000 (number of siloxane units *n* = 1, 9, 65, and 335), respectively (Figure 1a). For this purpose, mixtures of dry CH_3_CN and CH_2_Cl_2_ were chosen in different molar ratios ranging from 1:0 to 1:4 by volume to dissolve both APDMSs and ethynylcobaltocenium hexafluorophosphate yielding homogeneous mixtures (Table 1). The hydroamination was carried out at 40 °C under argon for 48 h to achieve high conversions of amino groups (see Section 2.3). The reaction was monitored using ^1^H, ^13^C{^1^H}, and ^29^Si{^1^H} NMR spectroscopy (Figures S2–S9).

During the reaction, two quasi-doublets at *δ* = 5.00 and 7.50 ppm with a coupling constant of 13.5 Hz appeared in ^1^H NMR spectra corresponding to the formation of a *trans*-isomer NHC*H*=C*H*Cc in the cases of cobaltocenium-terminated disiloxane Cc-APTMDS and Cc-APDMSs. A new broad signal at *δ* = 6.40–6.50 ppm corresponding to enamine proton N*H*CH=CHCc was also detected. In addition, the multiplet signal of protons of the CH_2_N group shifted from *δ* = 3.27 to 3.13 ppm in acetone-*d*_6_ and from 2.69 to 3.05 ppm in CDCl_3_ (Figures S2a, S3a, S4a, S5a, S6a, S7a, S8a and S9a), indirectly indicating the occurrence of hydroamination. These data are in full agreement with previously reported hydroamination in refs [30,31].

The number-average molecular weights of the target Cc-APDMSs were also determined by ^1^H NMR end-group analysis. Thus, the *M_n_* was approx. 964, 1630, 5860, and 23,150 in the case of Cc-APTMDS, Cc-APDMS850, Cc-APDMS5000, and Cc-APDMS25000, respectively. However, it was shown that an increase in the *M_n_* of the pristine APDMS from 850 to 25,000 contributes to the hindrance of its modification by ethynylcobaltocenium. The percentage of reacted amino groups was determined as 85%, 77%, and 45% for Cc-APDMS850, Cc-APDMS5000, and Cc-APDMS25000, respectively. The main reason is the steric factor reducing the accessibility of the terminal amino group.

In the ^13^C{^1^H} NMR spectra of Cc-APTMDS and Cc-APDMSs (freshly synthesized), the appearance of new signals was observed at *δ* = 83.8–83.9 and 144.7–146.4 ppm, corresponding to the carbon atoms of enamine (Figures S2b, S3b, S4b, S5b, S6b and S7b). In the ^29^Si{^1^H} NMR spectra of the polymers, signals of Si atoms of the polymer backbone (D) and signals of terminal groups (M^Cc^) were detected at *δ* = −22.0 (D), −21.3 (D), and 7.6 ppm (M^Cc^), correspondingly (Figures S4c, S5c, S6c, S7c, S8b and S9b). Conversely, a ^29^Si{^1^H} NMR spectrum of Cc-APTMDS shows a signal at *δ* = 7.8 ppm, which differs from one of pristine APTMDS (Figures S2c and S3c). The ^19^F{^1^H} and ^31^P{^1^H} NMR spectra confirm that anion PF_6_^−^ is present in Cc-APDMSs and does not contribute to the hydroamination reaction without noticeable changes in chemical shifts (Figures S2d,e, S3d,e, S4d,e, S5d,e, S6d,e and S7d,e).

The HRMS spectroscopy additionally confirmed the formation of cobaltocenium-terminated siloxanes. Thus, for disiloxane Cc-APTMDS, a molecular ion peak of the corresponding dication [C_34_H_48_Co_2_N_2_OSi_2_]^2+^ (*m*/2 = 337.0997) was identified with its characteristic isotopic distribution (Figure S10).

**Figure 2:**
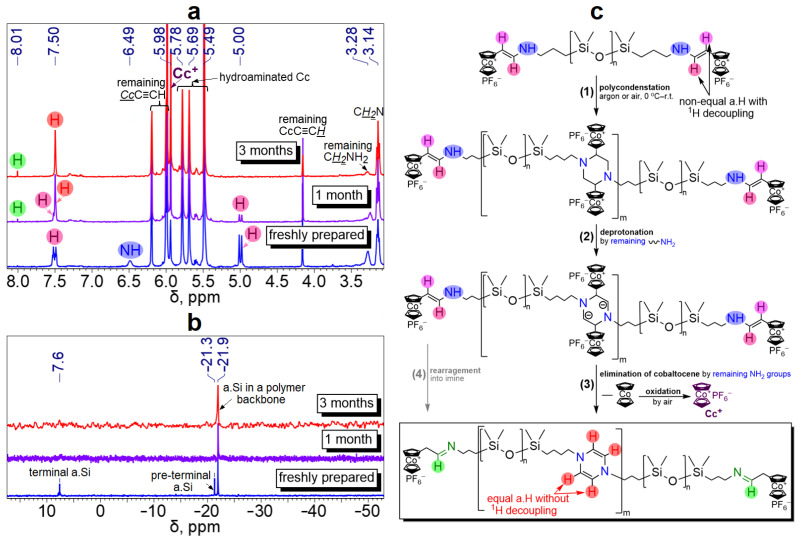
Changes in ^1^H NMR of P(Cc-AMS-*co*-DMS) when being stored in acetone-*d*_6_ in air (**a**), and changes in ^29^Si{^1^H} NMR of Cc-APDMS850 when being stored in acetone-*d*_6_ in air (**b**), plausible mechanism of condensation reaction between enamine groups in cobaltocenium-terminated (co)polymers (**c**).

A copolysiloxane containing cobaltocenium as side groups (P(Cc-AMS-*co*-DMS)) was also synthesized by hydroamination of the corresponding amino-comprising copolysiloxane with ethynylcobaltocenium hexafluorophosphate via the same procedure and conditions (see Section 2.3 and Figure 1b). The most noticeable difference between the initial substances and the target copolymer was established by ^1^H NMR showing the appearance of two doublets at *δ* = 5.00 and 7.51 ppm, a broad singlet of enamine (N*H*CH=CHCc) at *δ* = 6.49 ppm, and a shift of CH_2_ multiplets of the CH_2_CH_2_CH_2_ linker (Figure 2a, Figures S11a and S12a). The conversion of amino groups was estimated at 67%. The ^1^H NMR-based *M_n_* of P(Cc-AMS-*co*-DMS) was approx. 7930. The ^13^C{^1^H} and ^29^Si{^1^H} NMR spectra additionally confirmed the emergence of enamine side groups (Figures S11b,c and S12b,c). The ^19^F{^1^H} and ^31^P{^1^H} NMR spectra of P(Cc-AMS-*co*-DMS) were similar to the ones of the cobaltocenium-terminated Cc-APDMSs (Figures S11d,e and S12d,e).

The XPS spectra were acquired to determine the cobalt content in the obtained cobaltocenium-containing (poly)siloxanes (Figure S13). Thus, the cobalt content in Cc-APDMSs and P(Cc-AMS-*co*-DMS) varied from 0.3 (Cc-APDMS25000) to 2.0 wt.% (Cc-APDMS850) as presented in Table S1.

Hence, the polymer containing the largest number of cobaltocenium units (Cc-APDMS850) was characterized by FTIR and UV–vis spectroscopies. The FTIR spectrum (Figure S14a) contained bands of C=C stretching (*ν* = 1623 cm^−1^) and N– H stretching (*ν* = 3437 cm^−1^). In the UV–vis spectrum of Cc-APDMS850 in CH_3_CN (Figure S14b), there were peaks at *λ_max_* = 415 nm, attributed to the *d*– *d* transition, and at *λ_max_* = 481 nm, combining the *d*– *d* transition and LMCT of cobaltocenium, as in the case of reported cobaltocenium derivatives [30].

### 3.2. “Gelation” and Redox Properties of Cobaltocenium-Containing Polysiloxanes

It was found that hydroamination occurs with a “gelation” of the resulting cobaltocenium-containing polysiloxanes. All the cobaltocenium-containing (co)polysiloxanes were brown and red oils, whose mixtures thicken and “gelate” over one week, when stored both in air and an argon atmosphere at r.t. The obtained polymers form high-quality films (Figure S15). Apparently, this is due to the polymer chain lengths increasing and/or cross-linking, caused by the reaction between the two terminal enamine-cobaltocenium groups. According to refs. [34,35], enamines can condense into 1,4-dihydropyrazine derivatives with possible cobaltocenium elimination. It was shown that the NMR spectra (^1^H and ^29^Si{^1^H}) of Cc-APDMS850 and P(Cc-AMS-*co*-DMS) solutions in acetone-*d*_6_ changed significantly after storage in air at r.t. during 1–3 months (Figure 2a,b). After 3 months, they stopped changing.

For instance, in ^1^H NMR of P(Cc-AMS-*co*-DMS), the signals of enamine (N*H*CH=CHCc, *δ* = 6.49 ppm) and H atom from double bond (*trans*-NHCH=C*H*Cc, *δ* = 5.00 ppm) completely disappeared after 3 months. A doublet of another H atom from the double bond (*trans*-NHC*H*=CHCc, *δ* = 7.50 ppm) transformed into a singlet with a noticeable increase in intensity. An imine formation (*δ* = 8.01 ppm) and a rise of the singlet at *δ* = 5.98 ppm corresponding to free cobaltocenium cation (Cc^+^) were also detected, indicating cobaltocenium elimination (Figure 2a). Similar changes in ^1^H NMR were also true for Cc-APDMS850 when stored in air. In the ^29^Si{^1^H} NMR spectra of Cc-APDMSs, we can see a disappearance of a signal (M^Cc^) corresponding to a terminal Si atom, indirectly indicating a polycondensation reaction (Figure 2b). There is an observable loss of N–H stretching at 3437 cm^−1^ and a decrease in the intensity of the LMCT band at 481 nm in the FTIR and UV–vis spectra of Cc-APDMS850, respectively (Figure S14). However, there were no notable changes in the XPS spectra during storage of Cc-PDMSs (Figure S13), thereby showing that the ligand cyclopentadienyl environment of cobalt had not changed. Hence, all the described spectra were fully consistent with the condensation of enamines into 1,4-dihydropyrazine presented in Figure 2c and in the literature [34,35]. The mechanism of the condensation was identical for both cobaltocenium-terminated polysiloxanes and polysiloxanes containing cobaltocenium in the main chain.

In addition, the growth in the average molecular weight of the obtained (co)polymers during storage was established by sedimentation velocity experiments using an analytical ultracentrifuge. The corresponding molecular mass distributions are shown in Figure S16. The distributions were obtained via the *c*(*S*) and *c*(*M*) analysis in Sedfit. Such models, despite their spherical approximation, have been shown to provide adequate and physically sound values of the molecular masses for various polymer structures [36]. In the case of Cc-APDMS850, the main fraction was accompanied by the higher-molecular-mass tail showing the appearance of some fractions’ *M_SD_* ranges from 2000 to 7000 (Figure S16a), which, presumably, can be associated with a formation of a “condensed” polymer via the reaction between enamines (Figure 2c). The presence of high-molecular-weight fractions (*M_SD_* = 25,000– 200,000) was also observed for other synthesized Cc-PDMSs (Figure S16b). Particularly interesting was Cc-APDMS25000, showing a typical polydisperse-like distribution with the main fraction at *M_SD_* = 30,000 (approx. 80%) and a long high-molecular-mass tail with the molecular masses up to 200,000 (Figure S16b). As a result, it could be concluded that Cc-APDMSs and P(Cc-AMS-*co*-DMS) increased their molecular weight (for Cc-APDMS850, Cc-APDMS5000 and P(Cc-AMS-*co*-DMS) by approx. 3, 30, and 20 times, respectively) during storage both at r.t. (for 1–3 months) and 0 °C (for 3 months) by the reaction between enamine groups with the probable elimination of the free cobaltocenium cation Cc^+^.

As distinguished from typical polydimethylsiloxanes and Cc-APDMS25000, other obtained cobaltocenium-containing (poly)siloxanes in both forms (before and after being stored for 3 months) were highly soluble in most polar solvents, such as CH_3_CN, CH_3_OH, and acetone. After “gelation” all the obtained cobaltocenium-containing polysiloxanes formed high-quality red films over one week (Figure S15).

**Figure 3:**
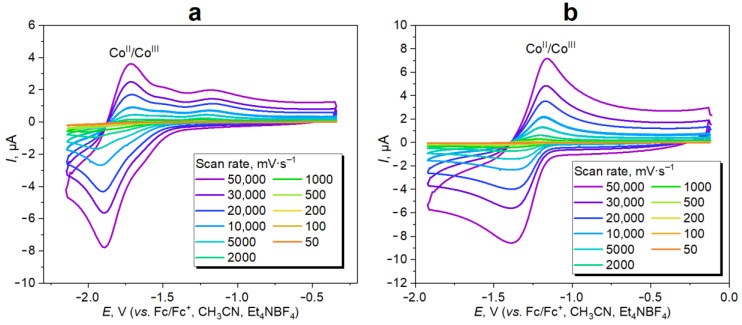
Cyclic voltammograms of Cc-APDMS850: freshly synthesized (**a**) and after being stored in air for 3 months (**b**), recorded in 0.1 M Et_4_NBF_4_ CH_3_CN solution at potential scan rates of 0.05–50 V·s^−1^.

The redox properties of Cc-PDMSs were confirmed by CV. To obtain a detectable electrochemical response, CV was carried out for Cc-APDMS850 with the highest metal content among the investigated polymers (2.0 wt.%, Table S1) in CH_3_CN solution using a classical three-electrode system under an argon atmosphere. The CV measurements were conducted with various potential scan rates from 0.05 to 50 V·s^−1^ within the potential range from −1.8 to 0 V vs. Ag|AgNO_3_ (−0.125 V vs. Fc/Fc^+^) using 0.1 M Et_4_NBF_4_ CH_3_CN as an electrolyte (Figure 3). In the obtained CVs of the freshly synthesized Cc-APDMS850 (Figure 3a), one pair of reversible oxidation/reduction peaks was observed at *E*_1/2_ ≈ −1.8 V, which is associated with the Co^II^/Co^III^ transition. The redox activity of the polymer remained at a good level after its storage for 1–3 months at r.t. (Figure 3b), but the redox potential shifted to *E*_1/2_ ≈ −1.3 V due to structural changes in the polymer chains described above (Figure 2). The corresponding Co^II^/Co^III^ redox potential was similar to that of other cobaltocenium-containing polymers reported in refs [16,28,30,37]. This fact confirms that the redox centers remained in the polymer structure.

In summary, it can be said that, for the first time, Cc-PDMSs were found to “gelate”, increasing their average molecular weight by approximately 30 times, when stored at room temperature for one week. The formed “gelated” polymers retained their redox properties. The “gelated” Cc-PDMSs remained soluble in most polar solvents, making them suitable to form high-quality redox films by the method of re-dissolution and drop casting with subsequent evaporation of the solvent.

## 4. Conclusions

In conclusion, a simple catalyst-free approach to synthesize cobaltocenium-containing polysiloxanes has been proposed, which consists of the immobilization of an ethynylcobaltocenium hexafluorophosphate on a polysiloxane chain using the hydroamination reaction of its triple bond with terminated or side amino groups of the (co)polysiloxanes. Remarkably, the proposed synthetic method has a number of advantages, namely that (*i*) it is catalyst-free (especially, it does not require expensive and unstable catalysts), (*ii*) the hydroamination reaction is fairly easy to carry out by mixing the reagents, (*iii*) the synthesis of Cc-PDMSs promotes an increase in the molecular weight (“gelation”) of the polymeric products, and (*iv*) the reaction products do not need to be purified from catalyst residues. The CV curves of the synthesized Cc-PDMSs contained one pair of reversible oxidation/reduction peaks (*E*_1/2_ ≈ −1.8 V), which is associated with the Co^II^/Co^III^ transition. Obtained polysiloxanes “gelated” over one week due to the polycondensation reaction of enamine-cobaltocenium-containing polymer chains, when stored at r.t., but remained soluble in most polar solvents. After storage for 1–3 months at r.t., the “gelated” Cc-PDMSs retained their redox activity with *E*_1/2_ shifted to −1.3 V. This approach allows an increase in the molecular weights of the synthesized polysiloxanes functionalized with redox-active cobaltocenium groups easily by 30 times, which improves their film-forming ability. Obtained redox-active Cc-APDMSs and P(Cc-AMS-*co*-DMS) can find potential application as components of batteries and other electronic and optoelectronic devices [1,15,23,24,27,28].

## Figures and Tables

**Table 1:** Amounts of reagents for synthesis of Cc-PDMSs and molecular weight characteristics of the resulting Cc-PDMSs.

Abbreviations of (Poly)siloxanes		Mass of Used Amino-Containing (Poly)siloxane, g	Used solvents		Characteristics **	
Cobaltocenium- Containing (Poly)siloxane	Used Amino- Containing (Poly)siloxane		CH_3_CN/CH_2_Cl_2_ Ratio, *V*/*V*	Volume, * mL	*M_n_*	Number of –[SiO]– Units
Cc-APTMDS	APTMDS	0.03	1:0	1.5	965	1
Cc-APDMS850	APDMS850	0.12	1:1	1.5	1630	9
Cc-APDMS5000	APDMS5000	0.70	1:4	3.5	5850	65
Cc-APDMS25000	APDMS25000	3.50		21.0	23,150	335
P(Cc-AMS-*co*-DMS)	P(AMS-*co*-DMS)	1.05		5.5	7930	96

* The volume of the CH_3_CN/CH_2_Cl_2_ mixture, which was used to dissolve an amino-containing polysiloxane; ** average molecular weight characteristics of the freshly obtained cobaltocenium-containing (poly)siloxanes based on ^1^H NMR.

## Data Availability

The data supporting this article have been included as a part of the Supplementary Materials.

## Supplementary Materials

The following supporting information can be downloaded at https://www.mdpi.com/article/10.3390/polym16202887/s1: Figure S1: Deciphered NMR spectra of ethynylcobaltocenium hexafluorophosphate registered in acetone-*d*_6_: ^1^H (**a**), ^13^C{^1^H} (**b**), ^19^F{^1^H} (**c**), and ^31^P{^1^H} (**d**). Figure S2: Deciphered NMR spectra of Cc-APTMDS registered in acetone-*d*_6_: ^1^H (**a**), ^13^C{^1^H} (**b**), ^29^Si{^1^H} (stacked with pristine APTMDS) (**c**), ^19^F{^1^H} (**d**), ^31^P{^1^H} (**e**), and its chemical formula (**f**). Figure S3: Full NMR spectra of Cc-APTMDS registered in acetone-*d*_6_: ^1^H (**a**), ^13^C{^1^H} (**b**), ^29^Si{^1^H} (**c**), ^19^F{^1^H} (**d**), and ^31^P{^1^H} (**e**). Figure S4: Deciphered NMR spectra of Cc-APDMS850 registered in acetone-*d*_6_: ^1^H (**a**), ^13^C{^1^H} (**b**), ^29^Si{^1^H} (**c**), ^19^F{^1^H} (**d**), ^31^P{^1^H} (**e**), and its chemical formula (**f**). Figure S5: Full NMR spectra of Cc-APDMS850 registered in acetone-*d*_6_: ^1^H (**a**), ^13^C{^1^H} (**b**), ^29^Si{^1^H} (**c**), ^19^F{^1^H} (**d**), and ^31^P{^1^H} (**e**). Figure S6: Deciphered NMR spectra of Cc-APDMS5000 registered in acetone-*d*_6_: ^1^H (**a**), ^13^C{^1^H} (**b**), ^29^Si{^1^H} (**c**), ^19^F{^1^H} (**d**), ^31^P{^1^H} (**e**), and its chemical formula (**f**). Figure S7: Full NMR spectra of Cc-APDMS5000 registered in acetone-*d*_6_: ^1^H (**a**), ^13^C{^1^H} (**b**), ^29^Si{^1^H} (**c**), ^19^F{^1^H} (**d**), and ^31^P{^1^H} (**e**). Figure S8: Deciphered NMR spectra of Cc-APDMS25000 registered in CDCl_3_: ^1^H (**a**), ^29^Si{^1^H} (**b**), and its chemical formula (**c**). Figure S9: Full NMR spectra of Cc-APDMS25000 registered in acetone-*d*_6_: ^1^H (**a**) and ^29^Si{^1^H} (**b**). Figure S10: HRMS spectrum of Cc-APTMDS recorded in a positive-ion mode. Figure S11: Deciphered NMR spectra of P(Cc-AMS-*co*-DMS) registered in acetone-*d*_6_: ^1^H (**a**), ^13^C{^1^H} (**b**), ^29^Si{^1^H} (**c**), ^19^F{^1^H} (**d**), ^31^P{^1^H} (**e**), and its chemical formula (**f**). Figure S12: Full NMR spectra of P(Cc-AMS-*co-*DMS) registered in acetone-*d*_6_: ^1^H (**a**), ^13^C{^1^H} (**b**), ^29^Si{^1^H} (**c**), ^19^F{^1^H} (**d**), and ^31^P{^1^H} (**e**). Figure S13: XPS survey spectra ranging from 0 to 900 eV of original P(Cc-AMS-*co*-DMS) and after being stored for 1 month. Figure S14: Molecular spectra of Cc-APDMS850: FTIR-ATR spectra in the range of 400–4000 cm^–1^ (**a**) and UV–vis spectra in CH_3_CN solution in the range of 200– 800 nm (**b**). Figure S15: Photograph of Cc-APDMS5000 film on a glassy template after “gelation” during storage for one week. Figure S16: Molecular weight distributions of APDMS850 and Cc-APDMS850 (**a**) and other Cc-PDMSs (**b**) obtained by sedimentation velocity experiments of their solutions in acetone at r.t. Table S1: Concentrations of cobalt in the polymer samples.

## References

[1] Gracia R., Mecerreyes D. (2013). Polymers with Redox Properties: Materials for Batteries, Biosensors and More. Polym. Chem..

[2] Zhao C., Park J., Root S.E., Bao Z. (2024). Skin-Inspired Soft Bioelectronic Materials, Devices and Systems. Nat. Rev. Bioeng..

[3] Deriabin K.V., Islamova R.M. (2022). Ferrocenyl-Containing Oligosiloxanes and Polysiloxanes: Synthesis, Properties, and Application. Polym. Sci. Ser. C.

[4] Mark J.E., Schaefer D.W., Lin G. (2015). The Polysiloxanes.

[5] Yilgör E., Yilgör I. (2014). Silicone Containing Copolymers: Synthesis, Properties and Applications. Prog. Polym. Sci..

[6] Rashevskii A.A., Deriabin K.V., Parshina E.K., Islamova R.M. (2023). Self-Healing Redox-Active Coatings Based on Ferrocenyl-Containing Polysiloxanes. Coatings.

[7] Deriabin K.V., Vereshchagin A.A., Kirichenko S.O., Rashevskii A.A., Levin O.V., Islamova R.M. (2023). Self-Cross-Linkable Ferrocenyl-Containing Polysiloxanes as Flexible Electrochromic Materials. Mater. Today Chem..

[8] Nagarale R.K., Lee J.M., Shin W. (2009). Electrochemical Properties of Ferrocene Modified Polysiloxane/Chitosan Nanocomposite and Its Application to Glucose Sensor. Electrochim. Acta.

[9] Cazacu M., Vlad A., Marcu M., Racles C., Airinei A., Munteanu G. (2006). New Organometallic Polymers by Polycondensation of Ferrocene and Siloxane Derivatives. Macromolecules.

[10] Islamova R.M. (2016). Iron Compounds in Controlled Radical Polymerization: Ferrocenes, (Clathro)Chelates, and Porphyrins. Russ. J. Gen. Chem..

[11] Yu G., Suzaki Y., Abe T., Osakada K. (2013). Introduction of Ferrocene-Containing [2]Rotaxanes onto Siloxane, Silsesquioxane and Polysiloxanes via Click Chemistry. Dalton Trans..

[12] Martínez-Montero I., Bruña S., González-Vadillo A.M., Cuadrado I. (2014). Thiol–Ene Chemistry of Vinylferrocene: A Simple and Versatile Access Route to Novel Electroactive Sulfur- and Ferrocene-Containing Model Compounds and Polysiloxanes. Macromolecules.

[13] Inagaki T., Lee H.S., Skotheim T.A., Okamoto Y. (1989). Syntheses and Electrochemical Properties of Siloxane Polymers Containing Ferrocene and Dimethylferrocene. J. Chem. Soc. Chem. Commun..

[14] Zhao L., Liu X., Zhang L., Qiu G., Astruc D., Gu H. (2017). Metallomacromolecules Containing Cobalt Sandwich Complexes: Synthesis and Functional Materials Properties. Coord. Chem. Rev..

[15] Kocheva A.N., Deriabin K.V., Volkov A.I., Levin O.V., Islamova R.M. (2024). Cobaltocenium-Containing Polysiloxanes: Catalytic Synthesis, Structure, and Properties. ACS Appl. Polym. Mater..

[16] Connelly N.G., Geiger W.E. (1996). Chemical Redox Agents for Organometallic Chemistry. Chem. Rev..

[17] Baldaguez Medina P., Ardila Contreras V., Hartmann F., Schmitt D., Klimek A., Elbert J., Gallei M., Su X. (2023). Investigating the Electrochemically Driven Capture and Release of Long-Chain PFAS by Redox Metallopolymer Sorbents. ACS Appl. Mater. Interfaces.

[18] Liu X., Rapakousiou A., Deraedt C., Ciganda R., Wang Y., Ruiz J., Gu H., Astruc D. (2020). Multiple Applications of Polymers Containing Electron-Reservoir Metal-Sandwich Complexes. Chem. Commun..

[19] Li H., Yang P., Hwang J., Pageni P., Decho A.W., Tang C. (2022). Antifouling and Antimicrobial Cobaltocenium-Containing Metallopolymer Double-Network Hydrogels. Biomater. Transl..

[20] Yang P., Luo Y., Kurnaz L.B., Bam M., Yang X., Decho A.W., Nagarkatti M., Tang C. (2021). Biodegradable Polycaprolactone Metallopolymer–Antibiotic Bioconjugates Containing Phenylboronic Acid and Cobaltocenium for Antimicrobial Application. Biomater. Sci..

[21] Cuadrado I., Casado C.M., Lobete F., Alonso B., González B., Losada J., Amador U. (1999). Preparation and Redox Properties of Novel Polymerizable Pyrrole- and Allyl-Functionalized Cobaltocenium Monomers and Siloxane-Based Cobaltocenium Polymers. Organometallics.

[22] Wang Y., Jia X., Zhu M., Liu X., Chao D. (2020). Oligoaniline-Functionalized Polysiloxane/Prussian Blue Composite towards Bifunctional Electrochromic Supercapacitors. New J. Chem..

[23] Pionteck J., Wypych G. (2016). Handbook of Antistatics.

[24] Tan Y.J., Susanto G.J., Anwar Ali H.P., Tee B.C.K. (2021). Progress and Roadmap for Intelligent Self-Healing Materials in Autonomous Robotics. Adv. Mater..

[25] Yan J., Li X., Zhang X., Liu S., Zhong F., Zhang J., Zhang Q., Yan Y. (2022). Metallo-Polyelectrolyte-Based Waterborne Polyurethanes as Robust HCl Corrosion Inhibitor Mediated by Inter/Intramolecular Hydrogen Bond. ACS Appl. Polym. Mater..

[26] Liu S., Yan J., Shi J., Li X., Zhang J., Wang X., Cai N., Fang Q., Zhang Q., Yan Y. (2023). Rational Design of Cobaltocenium-Containing Polythioether Type Metallo-Polyelectrolytes as HCl Corrosion Inhibitors for Mild Steel. Polym. Chem..

[27] Wei J., Ren L., Tang C., Su Z. (2014). Electric-Stimulus-Responsive Multilayer Films Based on a Cobaltocenium-Containing Polymer. Polym. Chem..

[28] Beladi-Mousavi S.M., Sadaf S., Hennecke A., Klein J., Mahmood A.M., Rüttiger C., Gallei M., Fu F., Fouquet E., Ruiz J. (2021). The Metallocene Battery: Ultrafast Electron Transfer Self Exchange Rate Accompanied by a Harmonic Height Breathing. Angew. Chem. Int. Ed..

[29] Escorihuela J., Lledós A., Ujaque G. (2023). Anti-Markovnikov Intermolecular Hydroamination of Alkenes and Alkynes: A Mechanistic View. Chem. Rev..

[30] Wang Y., Rapakousiou A., Ruiz J., Astruc D. (2014). Metalation of Polyamine Dendrimers with Ethynylcobalticenium for the Construction of Mono- and Heterobimetallic Polycationic Metallodendrimers. Chem. Eur. J..

[31] Wang Y., Rapakousiou A., Latouche C., Daran J.-C., Singh A., Ledoux-Rak I., Ruiz J., Saillard J.-Y., Astruc D. (2013). Mild Uncatalyzed Hydroamination of an Electrophilic Alkyne, Ethynylcobalticinium. Chem. Commun..

[32] Vanicek S., Kopacka H., Wurst K., Müller T., Schottenberger H., Bildstein B. (2014). Chemoselective, Practical Synthesis of Cobaltocenium Carboxylic Acid Hexafluorophosphate. Organometallics.

[33] Brown P.H., Schuck P. (2006). Macromolecular Size-and-Shape Distributions by Sedimentation Velocity Analytical Ultracentrifugation. Biophys. J..

[34] Rodrigues A., Ferreira P.M.T., Monteiro L.S. (2004). Synthesis and Reactivity of a 1,4-Dihydropyrazine Derivative. Tetrahedron.

[35] Menia D., Pittracher M., Kopacka H., Wurst K., Neururer F.R., Leitner D., Hohloch S., Podewitz M., Bildstein B. (2023). Curious Case of Cobaltocenium Carbaldehyde. Organometallics.

[36] Perevyazko I., Gubarev A.S., Pavlov G.M. (2021). Analytical Ultracentrifugation and Combined Molecular Hydrodynamic Approaches for Polymer Characterization. Molecular Characterization of Polymers.

[37] Feuerstein A., Boßmann B., Rittner T., Leiner R., Janka O., Gallei M., Schäfer A. (2023). Polycobaltoceniumylmethylene—A Water-Soluble Polyelectrolyte Prepared by Ring-Opening Transmetalation Polymerization. ACS Macro Lett..

