# Supplementary material for: When a Side Reaction Is a Benefit: A Catalyst-Free Route to Obtain High-Molecular Cobaltocenium-Functionalized Polysiloxanes by Hydroamination
Source: Polymers (Basel). 2024 Oct 14;16(20):2887. doi: 10.3390/polym16202887 (PMC11510845; doi:10.3390/polym16202887)
Supplement: Supplementary file 1 [file polymers-16-02887-s001.zip › polymers-3225901-supplementary.pdf]

Supplementary Material

When a Side Reaction is a Benefit: A Catalyst-Free Route to Obtain High-Molecular Cobaltocenium-Functionalized Polysiloxanes by Hydroamination

Anastasia N. Kocheva[1,†], Konstantin V. Deriabin[1,†], Igor Perevyazko[2], Nadezhda A. Bokach[1,*], Vadim P. Boyarskiy[1,*], and Regina M. Islamova[1,*]

[1] Institute of Chemistry, St. Petersburg State University, 7/9 Universitetskaya nab., 199034 St. Petersburg, Russia; kocheva-nastya@mail.ru (A.N.K.); deriabin.k@yahoo.com (K.V.D.)
[2] Department of Molecular Biophysics and Polymer Physics, St. Petersburg State University, 7/9 Universitetskaya nab., 199034 St. Petersburg, Russia; igor.perevyazko@gmail.com (I.P.)
* Correspondence: n.bokach@spbu.ru (N.A.B.); v.boiarskii@spbu.ru (V.P.B.); r.islamova@spbu.ru (R.M.I.)
† These authors contributed equally to this work.

Content

S1. NMR and mass spectra S2

S1.1. NMR spectra of ethynylcobaltocenium hexafluorophosphate S2

S1.2. NMR and mass spectra of cobaltocenium-terminated di- and polysiloxanes S3

S1.3. NMR spectra of copolysiloxane containing cobaltocenium as side groups S11

S2. XPS data S13

S3. Molecular spectroscopic data and photographs S14

S4. Sedimentation velocity data S15

S1. NMR and mass spectra

S1.1. NMR spectra of ethynylcobaltocenium hexafluorophosphate

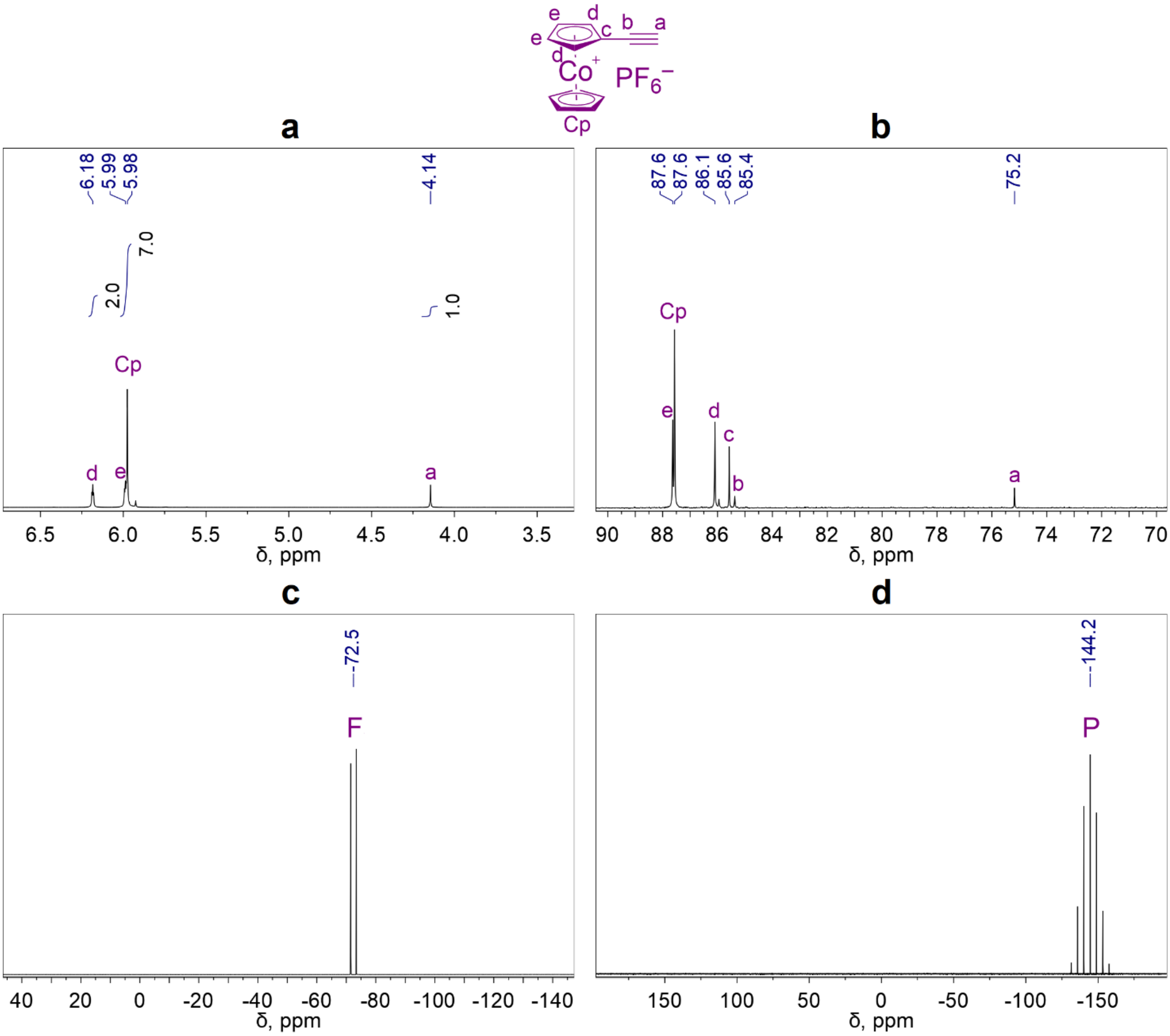

Figure S1. Deciphered NMR spectra of ethynylcobaltocenium hexafluorophosphate registered in acetone-d_6: ^{1}H (**a**), $^{13}C\{^{1}H\}$ (**b**), $^{19}F\{^{1}H\}$ (**c**), and $^{31}P\{^{1}H\}$ (**d**).

S1.2. NMR and mass spectra of cobaltocenium-terminated di- and polysiloxanes

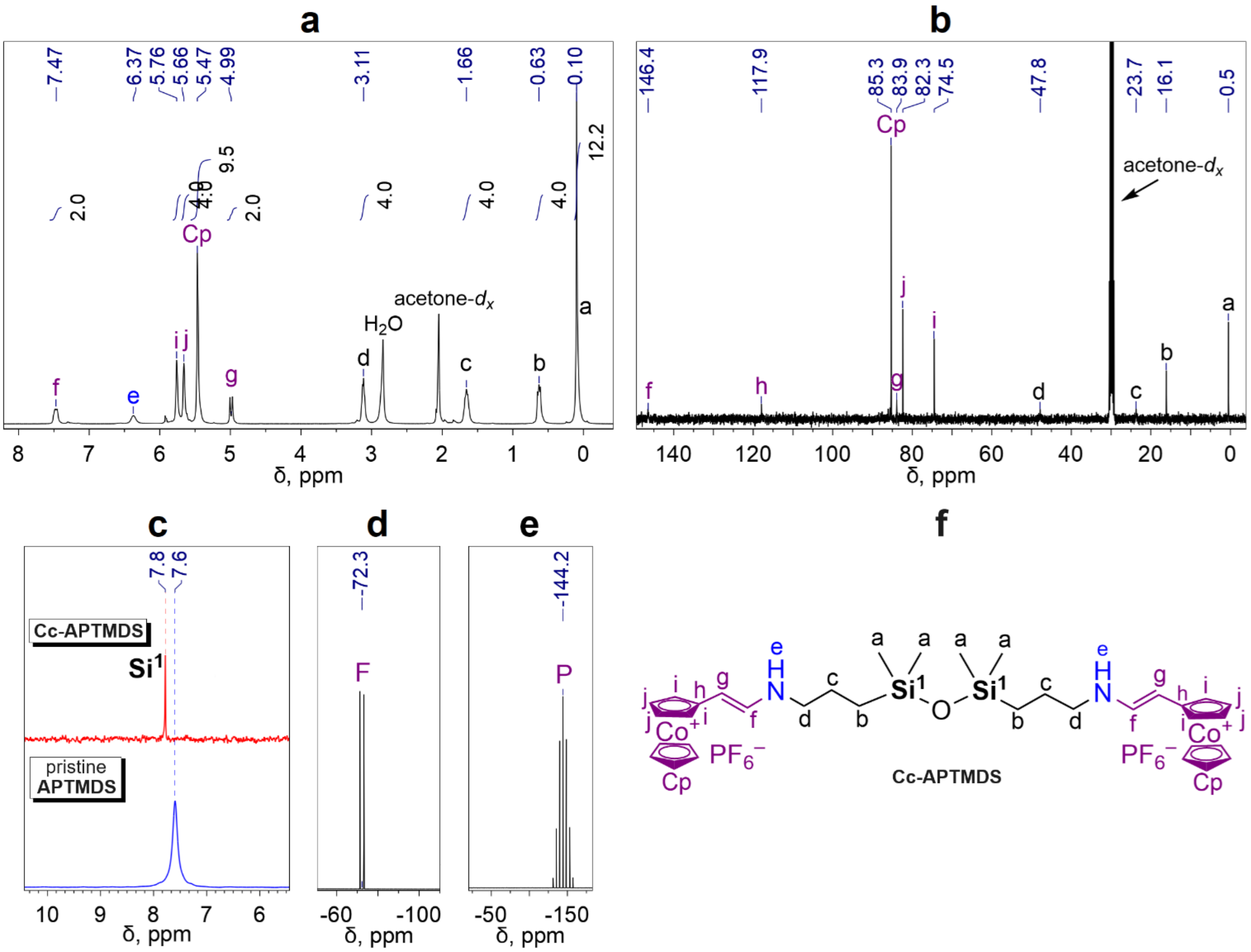

Figure S2. Deciphered NMR spectra of Cc-APTMDS registered in acetone-d_6: ^{1}H (**a**), ^{13}C{^{1}H} (**b**), ^{29}Si{^{1}H} (stacked with pristine APTMDS) (**c**), ^{19}F{^{1}H} (**d**), ^{31}P{^{1}H} (**e**), and its chemical formula (**f**).

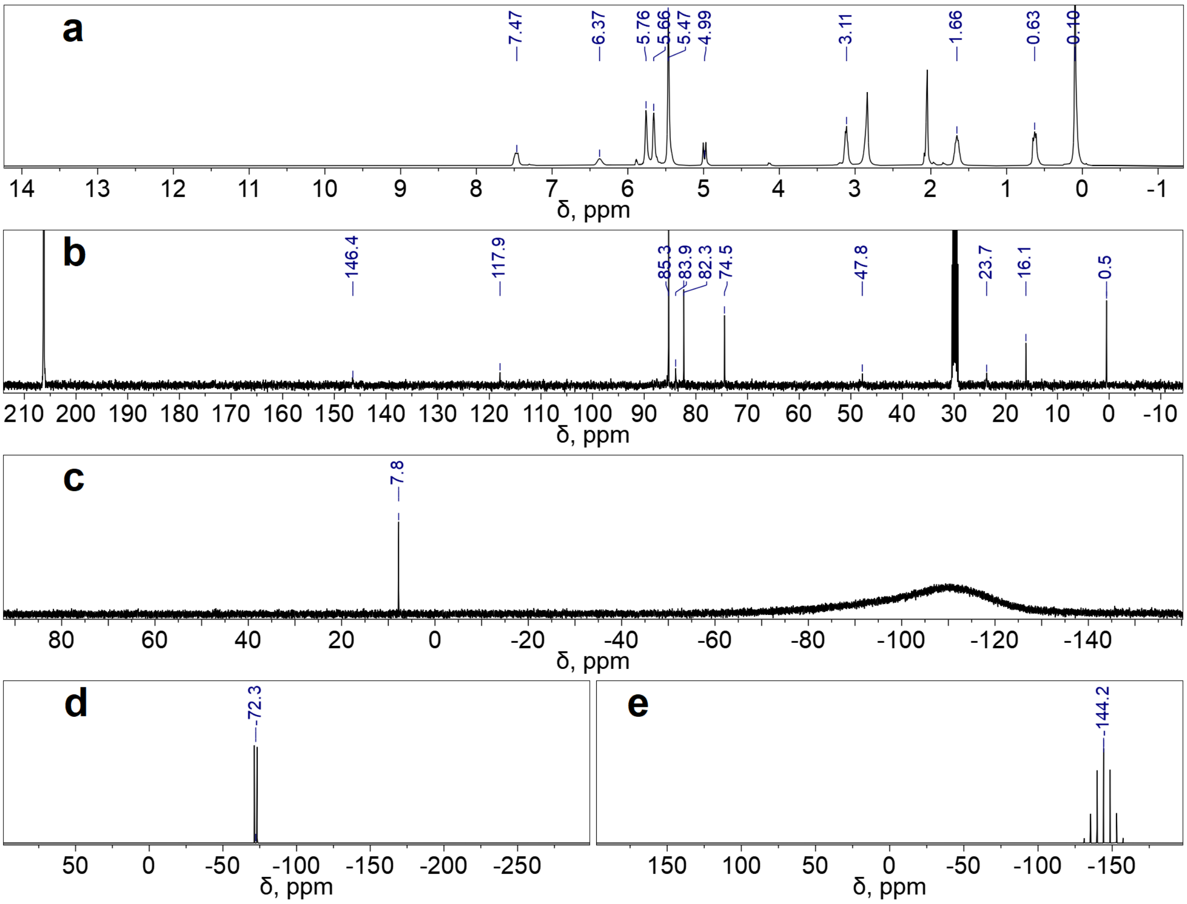

Figure S3. Full NMR spectra of Cc-APTMDS registered in acetone-d_6: ^{1}H (**a**), $^{13}C\{^{1}H\}$ (**b**), $^{29}Si\{^{1}H\}$ (**c**), $^{19}F\{^{1}H\}$ (**d**), and $^{31}P\{^{1}H\}$ (**e**).

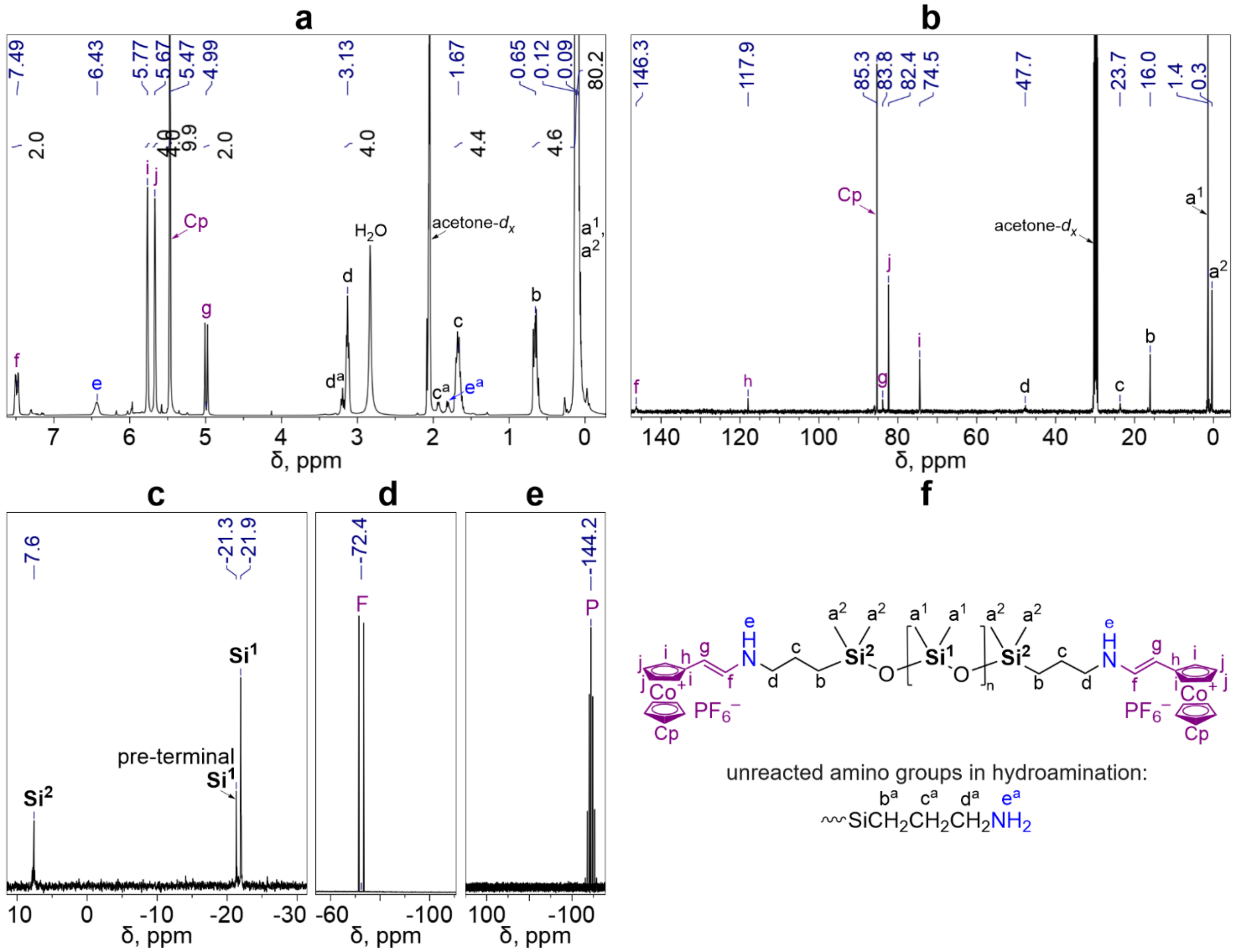

Figure S4. Deciphered NMR spectra of Cc-APDMS850 registered in acetone-d_6: ^{1}H (**a**), ^{13}C{^{1}H} (**b**), ^{29}Si{^{1}H} (**c**), ^{19}F{^{1}H} (**d**), ^{31}P{^{1}H} (**e**), and its chemical formula (**f**).

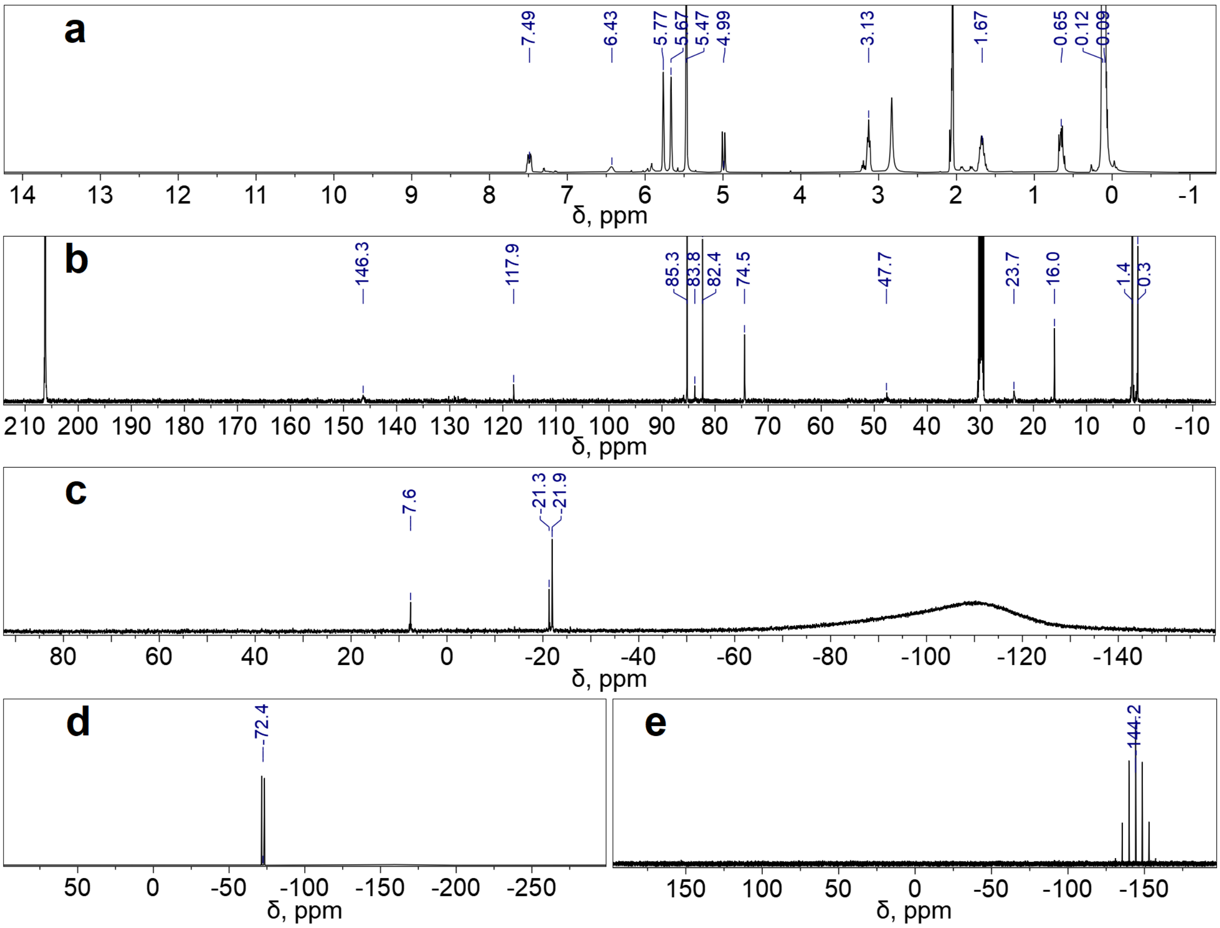

Figure S5. Full NMR spectra of Cc-APDMS850 registered in acetone-d_6: ^{1}H (**a**), ^{13}C{^{1}H} (**b**), ^{29}Si{^{1}H} (**c**), ^{19}F{^{1}H} (**d**), and ^{31}P{^{1}H} (**e**).

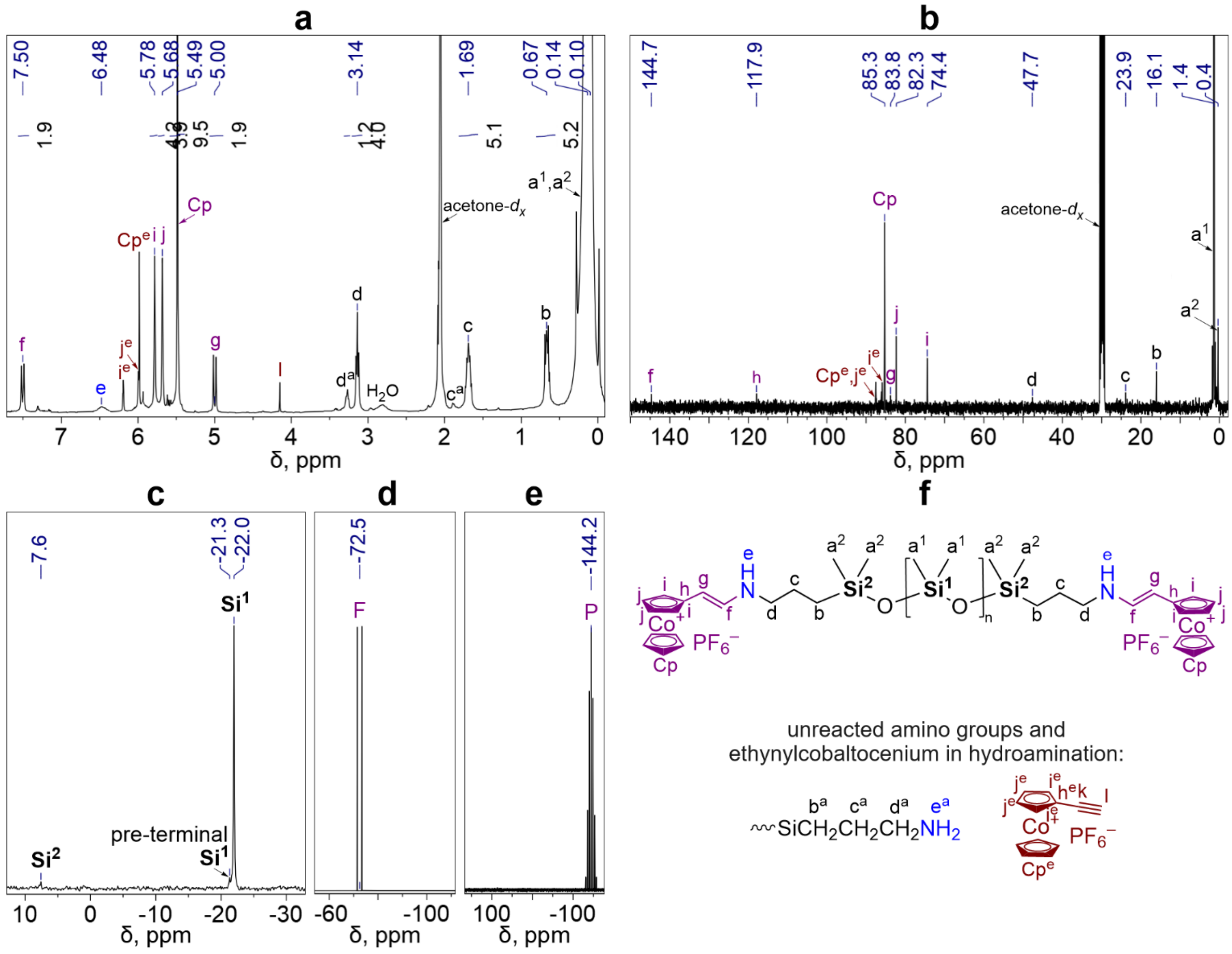

Figure S6. Deciphered NMR spectra of Cc-APDMS5000 registered in acetone-d_6: ^{1}H (**a**), ^{13}C{^{1}H} (**b**), ^{29}Si{^{1}H} (**c**), ^{19}F{^{1}H} (**d**), ^{31}P{^{1}H} (**e**), and its chemical formula (**f**).

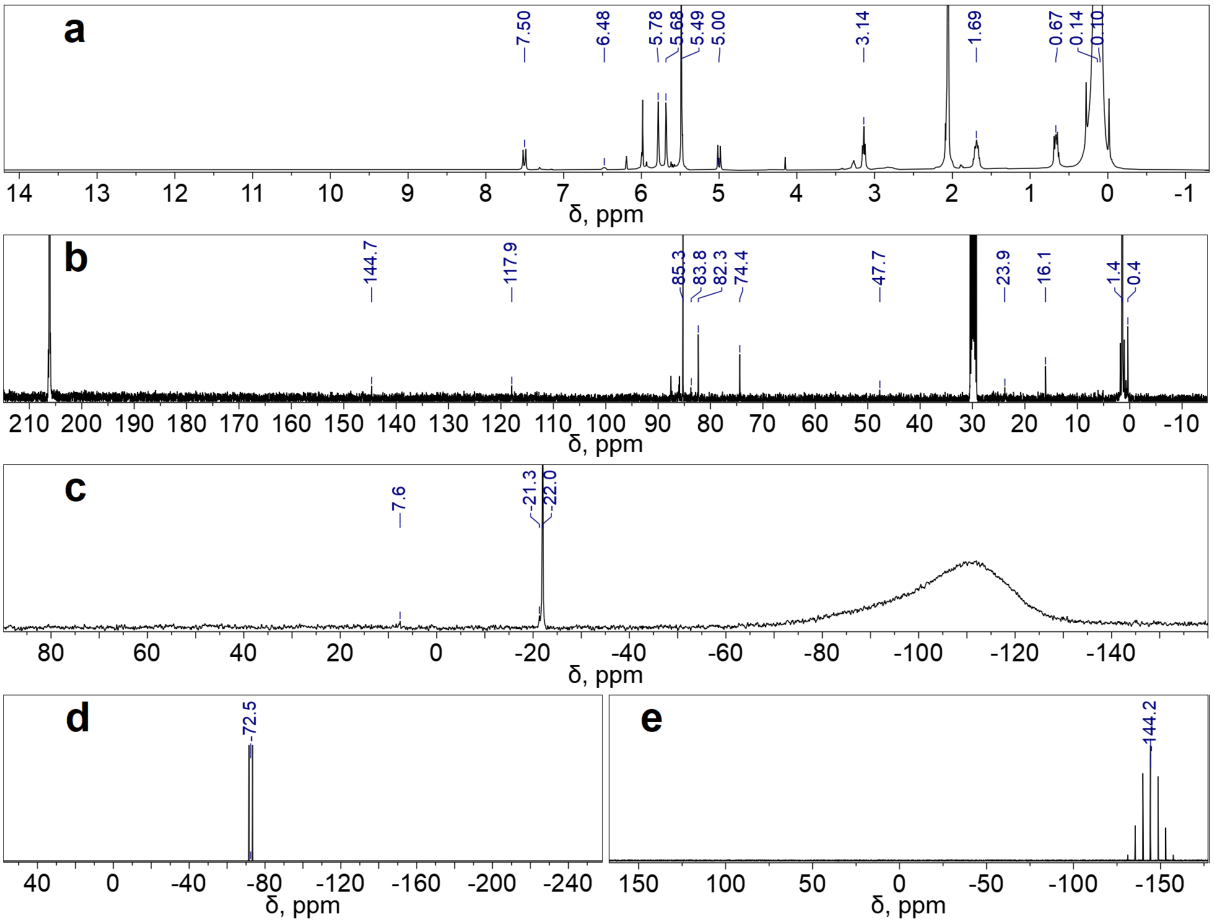

Figure S7. Full NMR spectra of Cc-APDMS5000 registered in acetone-d_6: ^{1}H (**a**), $^{13}C\{^{1}H\}$ (**b**), $^{29}Si\{^{1}H\}$ (**c**), $^{19}F\{^{1}H\}$ (**d**), and $^{31}P\{^{1}H\}$ (**e**).

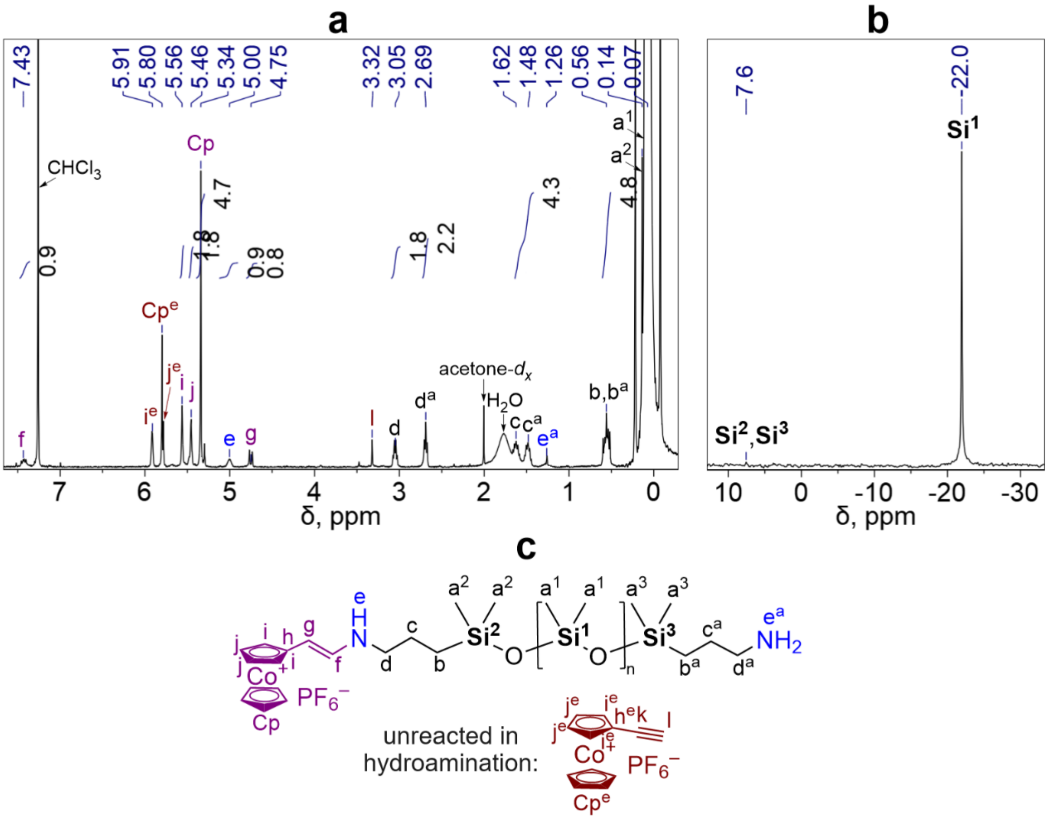

Figure S8. Deciphered NMR spectra of Cc-APDMS25000 registered in CDCl$_3$: ^{1}H (**a**), ^{29}Si{^{1}H} (**b**), and its chemical formula (**c**).

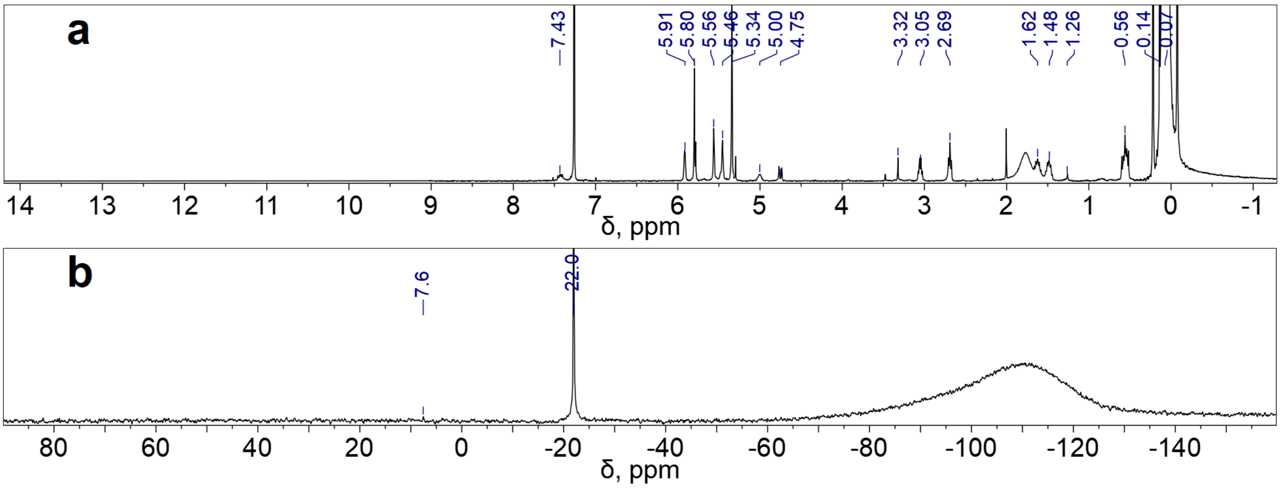

Figure S9. Full NMR spectra of Cc-APDMS25000 registered in acetone-d_6: ^{1}H (**a**) and ^{29}Si{^{1}H} (**b**).

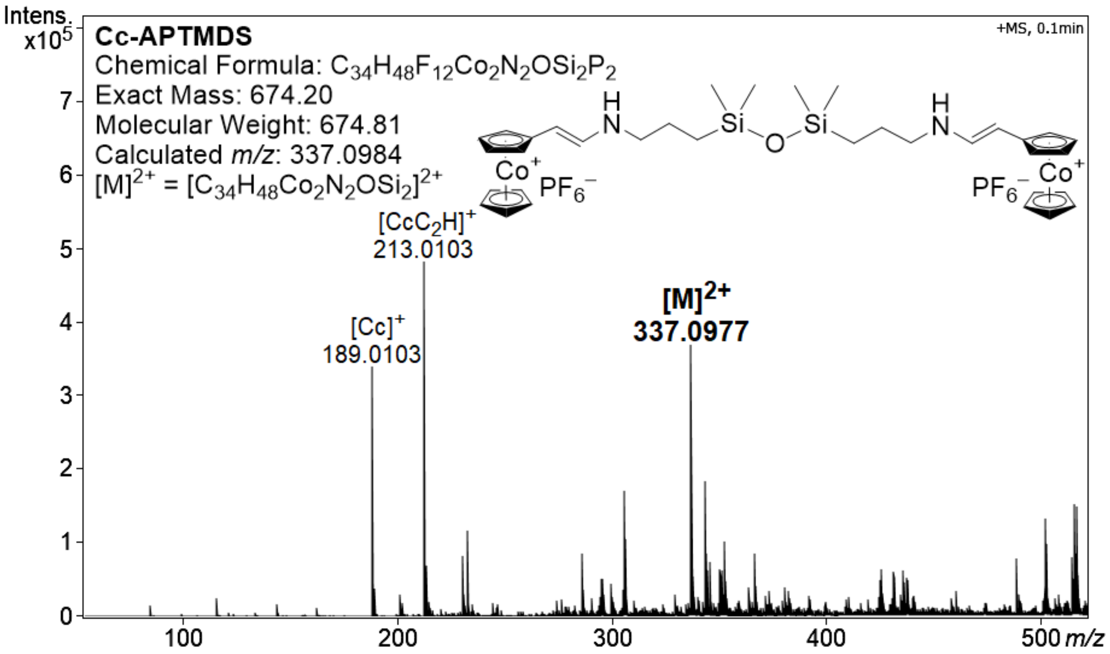

Figure S10. HRMS spectrum of Cc-APTMDS recorded in a positive ion mode.

S1.3. NMR spectra of copolysiloxane containing cobaltocenium as side groups

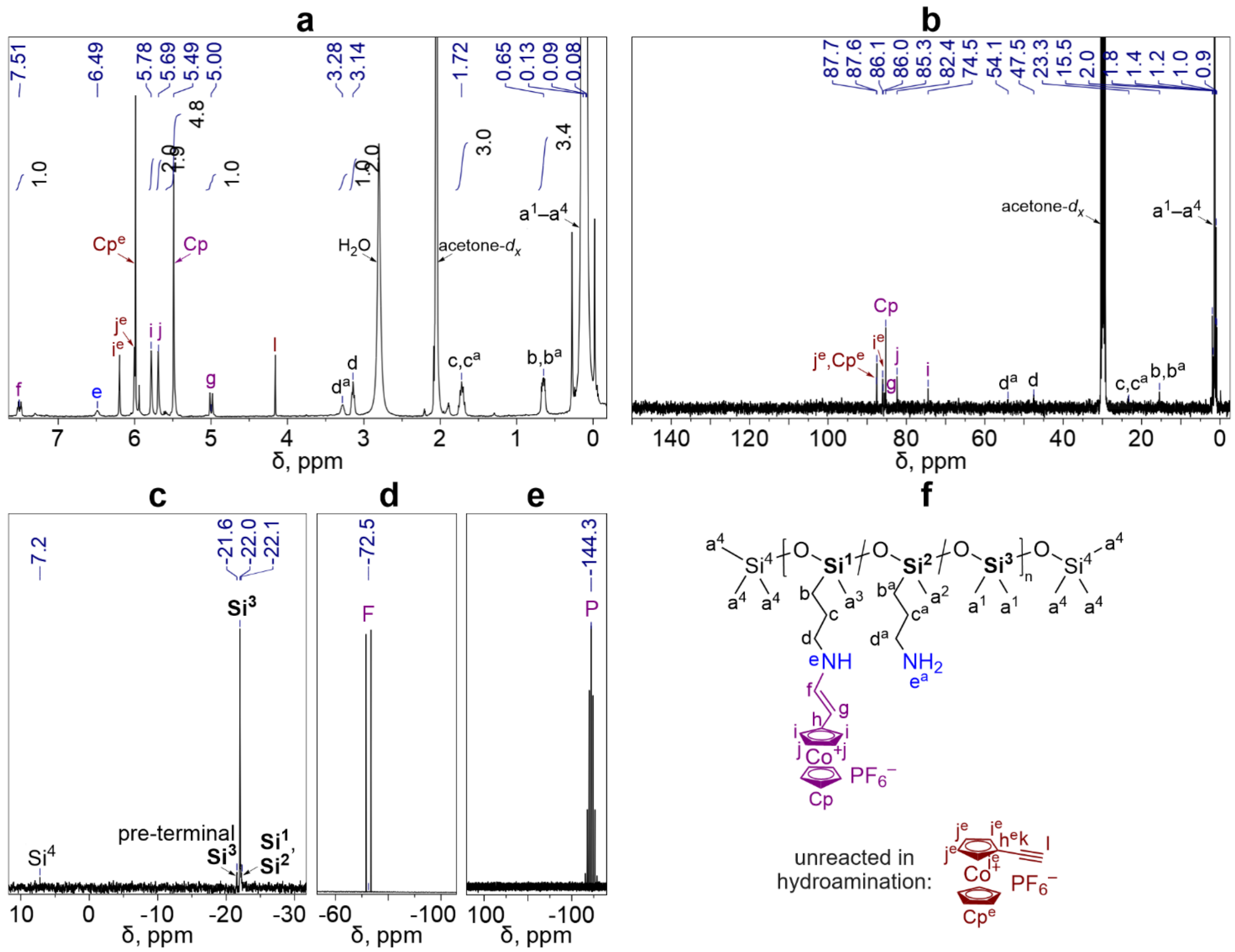

Figure S11. Deciphered NMR spectra of P(Cc-AMS-*co*-DMS) registered in acetone-*d*$_6$: ^{1}H (**a**), ^{13}C{^{1}H} (**b**), ^{29}Si{^{1}H} (**c**), ^{19}F{^{1}H} (**d**), ^{31}P{^{1}H} (**e**), and its chemical formula (**f**).

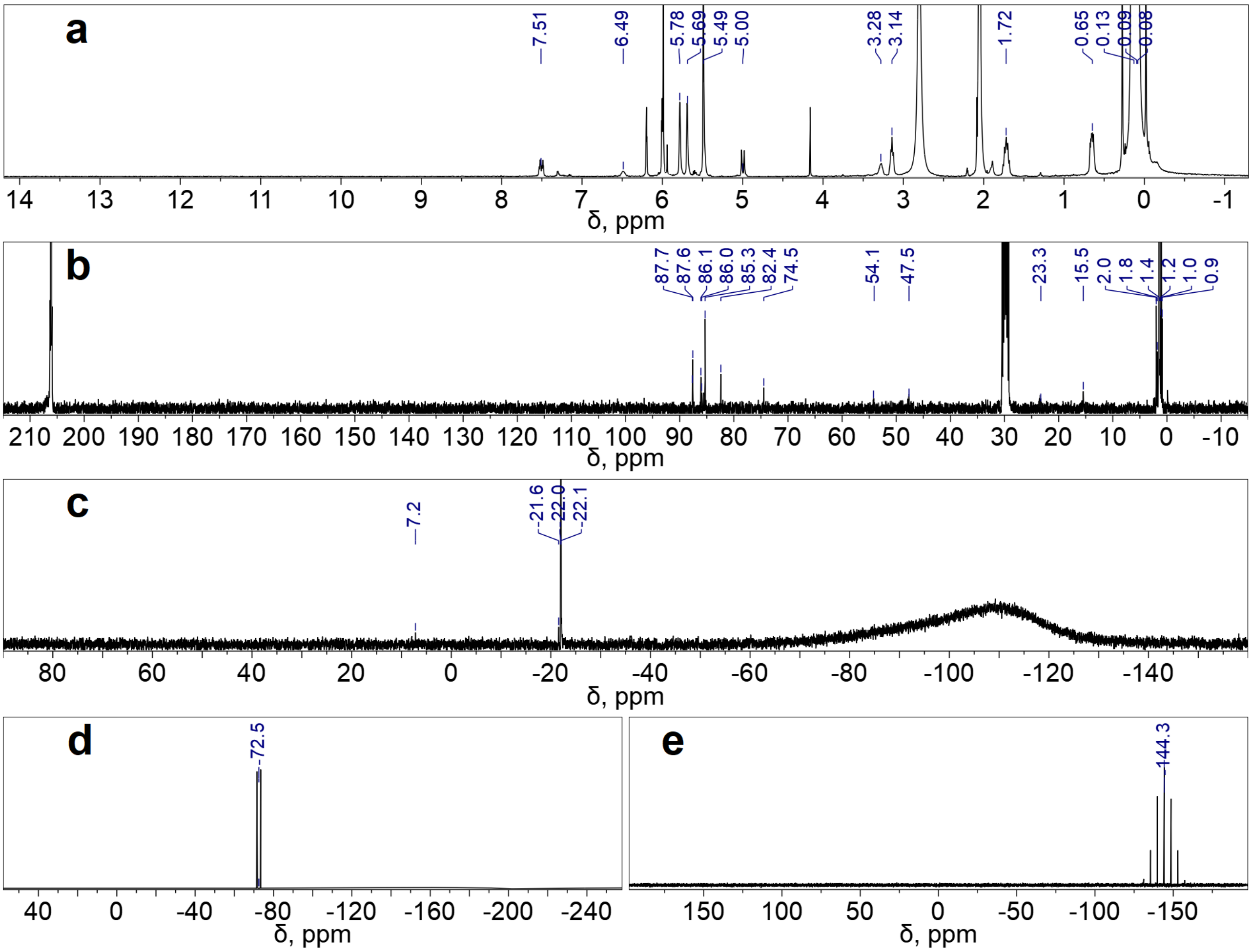

Figure S12. Full NMR spectra of P(Cc-AMS-*co*-DMS) registered in acetone-d_6: ^{1}H (**a**), $^{13}C\{^{1}H\}$ (**b**), $^{29}Si\{^{1}H\}$ (**c**), $^{19}F\{^{1}H\}$ (**d**), and $^{31}P\{^{1}H\}$ (**e**).

S2. XPS data

Table S1. Concentrations of cobalt in the polymer samples.

**Cobaltocenium-containing (poly)siloxane**	**Peak BE, [a] eV**	**FWHM [b]**	**Peak area**	**Concentration of cobalt**		**Concentration of cobaltocenium, wt.%**
				**Atomic %**	**wt. %**	
Cc-APTMDS	780.5	1.8	3442	1.4	5.2	15.8
Cc-APDMS850	780.8	1.8	1019	0.6	2.0	6.1
Cc-APDMS5000	779.5	0.3	253	0.2	0.6	1.8
Cc-APDMS25000	780.0	0.2	164	0.1	0.3	0.9
P(Cc-AMS-*co*-DMS)	779.6	0.7	336	0.2	0.5	1.5

[a] BE — binding energy; [b] FWHM — full width at half maximum.

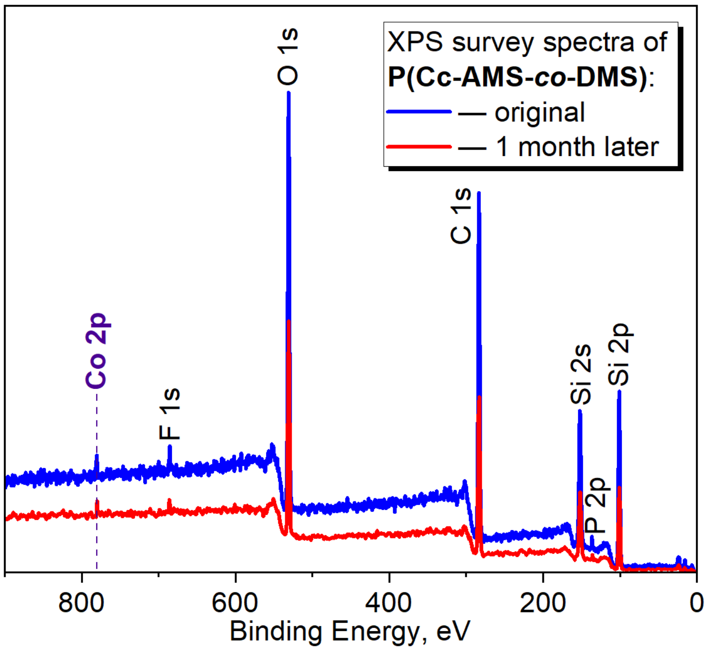

Figure S13. XPS survey spectra ranging from 0 to 900 eV of original (freshly synthesized) P(Cc-AMS-*co*-DMS) and after being stored for 1 month.

S3. Molecular spectroscopic data and photographs

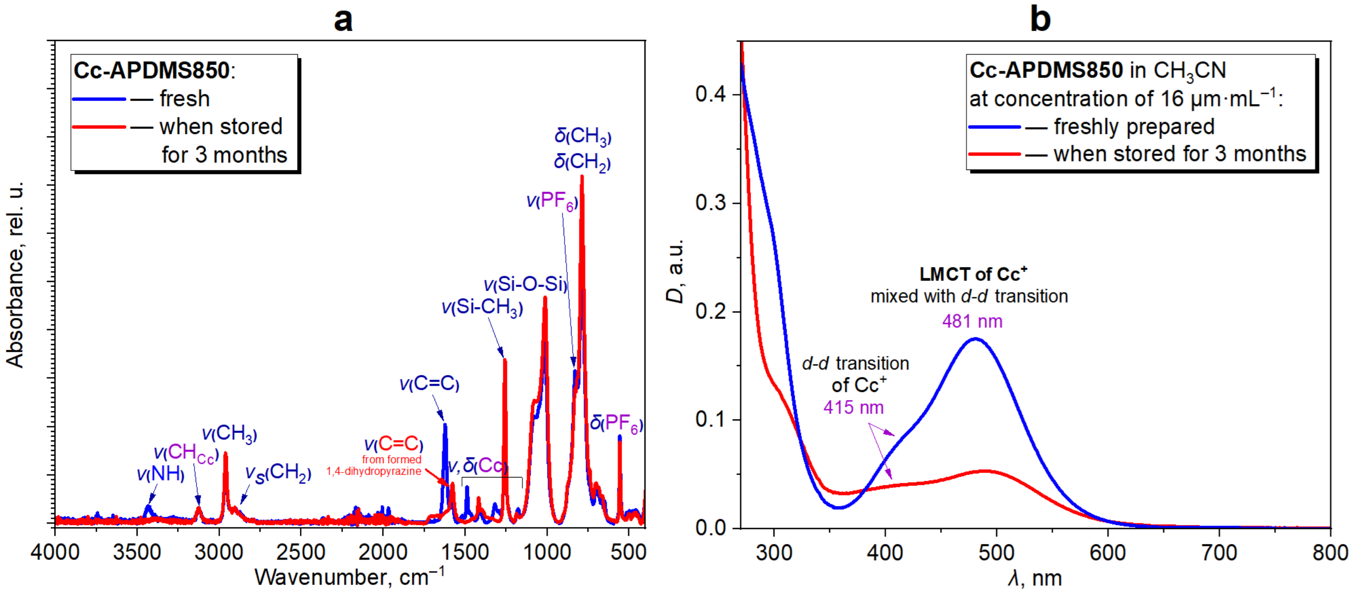

Figure S14. Molecular spectra of Cc-APDMS850: FTIR-ATR spectra in the range of 400–4000 cm^{-1} (**a**) and UV-vis spectra in CH_3CN solution in the range of 200–800 nm (**b**).

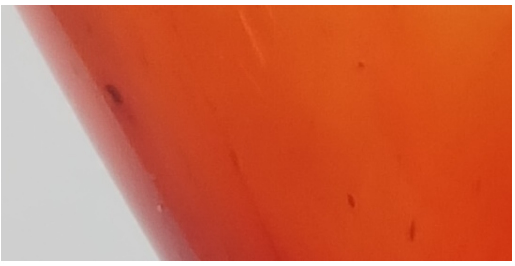

Figure S15. Photograph of Cc-APDMS5000 film on a glassy template after "gelation" during storage for one week.

S4. Sedimentation velocity data

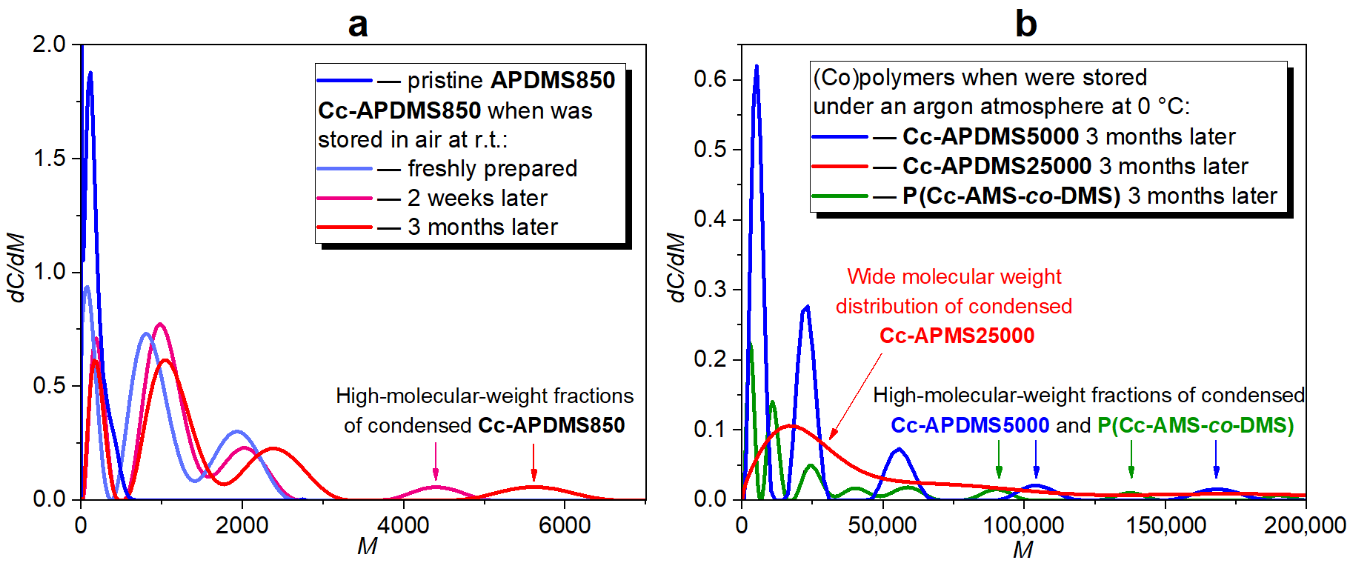

Figure S16. Molecular weight distributions of APDMS850 and Cc-APDMS850 (**a**) and other Cc-PDMSs (**b**) obtained by sedimentation velocity experiments of their solutions in acetone at r.t.
